# A revised model for PHF20L1 Tudor function: DNA binding overrides methylation selectivity on nucleosomes

**DOI:** 10.1016/j.jbc.2026.113181

**Published:** 2026-05-21

**Authors:** Xiaolei Huang, Qin Xiao, Xin Liu, Xinci Shang, Zhuowen Wang, Haiyun Hu, Yinhao Zhou, Qiuyan Huang, Tao Jiang, Su Qin, Yangtong Huang, Jia-Bin Li, Yanli Liu

**Affiliations:** 1 Jiangsu Key Laboratory of Drug Discovery and Translational Research for Brain Diseases, College of Pharmaceutical Sciences, Soochow University, Suzhou, Jiangsu, China; 2 Jiangsu Province Engineering Research Center of Precision Diagnostics and Therapeutics Development, Soochow University, Suzhou, Jiangsu, China; 3 College of Pharmaceutical Sciences, Suzhou International Joint Laboratory for Diagnosis and Treatment of Brain Diseases, Soochow University, Suzhou, Jiangsu, China; 4 Division of Life Sciences and Medicine, Department of Chemistry and the First Affiliated Hospital of USTC, School of Life Sciences and School of Biomedical Engineering, University of Science and Technology of China, Hefei, Anhui, China; 5 Life Science Research Center, Southern University of Science and Technology, Shenzhen, Guangdong, China

**Keywords:** histone binding, methyl-lysine, nucleosome binding PHF20L1, Tudor domain

## Abstract

Plant homeodomain finger protein 20-like protein 1 (PHF20L1) is a methyl-lysine reader that regulates chromatin remodeling and transcription *via* its tandem Tudor and PHD finger domains. Here, we characterize the selectivity of these Tudor domains using biophysical and structural approaches. Quantitative fluorescence polarization (FP) and isothermal titration calorimetry (ITC) reveal that Tudor1 binds to both mono- and di-methylated H3K36 and H4K20, with a modest preference for H3K36me1, whereas Tudor2 is highly specific for H4K20me2. Their tandem arrangement enhances affinity for methylated H3K36 through cooperative binding. The crystal structure of Tudor1 in complex with H3K36me1 reveals the basis for its preference: a deep, narrow aromatic cage formed by Y24, Y29, F47, W50, and Y54 accommodates the mono-methylammonium group, while D23 contributes a critical hydrogen bond that stabilizes the interaction. Mutations in this cage abolish H3K36me1 binding, and mutation of corresponding cage-forming residues (W97A/Y103A, based on our solved apo Tudor2 structure) disrupts Tudor2 recognition of H4K20me2. Structural comparison across Tudor family readers for H3K36me and H4K20me further indicates that the number and orientation of acidic residues within the otherwise hydrophobic aromatic cages fine-tune selectivity among methyl-lysine states. Unexpectedly, nucleosome-binding assays reveal that the tandem Tudor domains bind to DNA with high affinity and lose methylation selectivity, contrasting sharply with the peptide-level results. Thus, our findings revise the mechanistic framework for PHF20L1 chromatin engagement: from a selective methyl-lysine reader to a high-affinity DNA-binding module on nucleosomes.

In eukaryotes, genomic DNA wraps around histone octamers—each composed of two copies of H2A, H2B, H3, and H4—to form nucleosomes, which are further compacted into highly organized chromatin (1, 2). The N-terminal tails of histone undergo diverse post-translational modifications (PTMs), such as methylation, acetylation, phosphorylation, and ubiquitination (3). These PTMs establish a “histone code” that dynamically modulates chromatin conformation and DNA accessibility, thereby shaping transcription-factor binding and gene expression to mediate epigenetic control of cell fate without altering the underlying DNA sequence (4). Among histone PTMs, methylation is particularly prominent. Methyl groups are covalently added to lysine (K) or arginine (R) residues and can occur in mono- (Kme1), di- (Kme2), or tri-methylated (Kme3) states (4, 5). These marks are deposited and removed by histone methyltransferases (HMTs, “writers”) and demethylases (KDMs, “erasers”), and interpreted by “reader” proteins bearing MBT, chromodomain, Tudor, PWWP, PHD, WD40, ANK, or BAH domains (6, 7). By recruiting or stabilizing nuclear complexes at specific genomic loci, these readers regulate key processes such as transcription, RNA splicing, and DNA damage repair (7, 8, 9, 10, 11). Dysregulation of these readers is implicated in cancer (12, 13), immune disorders (14, 15), neurodegenerative and developmental diseases (15, 16), highlighting them as attractive targets for therapeutic intervention (17).

Plant homeodomain (PHD) finger protein 20-like protein 1 (PHF20L1) is a multidomain methyl-lysine reader belonging to the Tudor domain-containing proteins (TDRDs) family. It comprises two N-terminal Tudor domains (Tudor1 and Tudor2) and a C-terminal PHD domain (Fig. 1*A*). Through these modules, PHF20L1 recognizes methyl-lysine marks on histone and non-histone proteins and contributes transcriptional regulation, chromatin remodeling, and protein stabilization. For example, PHF20L1 recognizes monomethylated pRb (retinoblastoma-associated protein) at K810 (K810me1) *via* its Tudor1 domain, recruits the MOF (males absent on the first) complex to E2F target genes, and enhances H4K16 acetylation, thereby maintaining the pRb-dependent G1–S checkpoint and supporting cell-cycle progression (18). In addition to its role in cell cycle regulation, PHF20L1 also stabilizes key regulatory proteins by competing with degradation machinery. For instance, it binds to the monomethylated K142 site of DNMT1 (DNA (cytosine-5) methyltransferase 1) through Tudor1, blocking its recognition by L3MBTL3 (lethal(3)malignant brain tumor-like protein 3) and subsequent ubiquitin–proteasome degradation (19, 20). Similarly, PHF20L1 binds to monomethylated SOX2 at K42, protecting it from methylation-directed degradation and supporting tumor stemness (21). Notably, recent studies have revealed that the Tudor2 domain of PHF20L1 specifically recognizes H3K27me2 and recruits both the polycomb repressive complex 2 (PRC2) and the Mi-2/nucleosome remodeling and deacetylase (NuRD) complex to target chromatin regions. By coordinating PRC2-mediated methylation with NuRD-mediated deacetylation of H3K27, PHF20L1 enforces silencing of tumor-suppressor genes such as *BRCA1* and *HIC1*, thereby promoting breast cancer proliferation and metastasis (22). Due to these regulatory roles, aberrant PHF20L1 expression is strongly associated with several malignancies, including breast (23), ovarian (24) and colorectal cancers (25).

**Figure 1:**
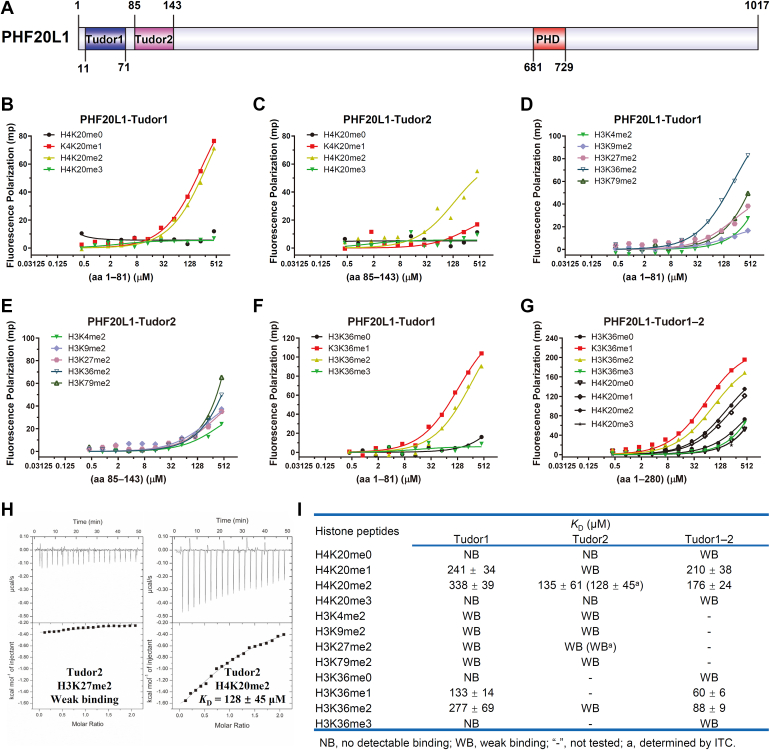
**Differential histone recognition by PHF20L1 Tudor domains.** Tudor1 preferentially binds to mono- and di-methylated H3K36 and H4K20, whereas Tudor2 specifically recognizes H4K20me2. *A*, Domain architecture of PHF20L1. *B–G*, FP binding curves for PHF20L1 Tudor1, Tudor2 and Tudor1–2 with the indicated histone peptides. *H*, ITC binding curves of PHF20L1 Tudor2 with H3K27me2 (*left*) and H4K20me2 (*right*). *I*, summary of binding affinities (*K*_D_) determined by FP or ITC. FP *K*_D_ values were obtained from duplicate measurements fitted to a one-site binding model in GraphPad Prism version 8 software. ITC *K*_D_ values were calculated from single experiments with the fitting error reported.

Motivated by the reported roles of PHF20L1 Tudor domains in methyl-lysine recognition, we systematically investigated the interactions between the human PHF20L1 Tudor modules and a panel of histone peptides carrying different lysine methylation states (Kme0–Kme3) using fluorescence polarization (FP) binding assays. FP measurements revealed that the PHF20L1 Tudor1 domain preferentially binds to H3K36me1 (*K*_D_ = 133 ± 14 μm), whereas a tandem Tudor1–2 module displays increased affinity for the same peptide (*K*_D_ = 60 ± 6 μm). In contrast, the Tudor2 domain preferentially recognizes H4K20me2 (*K*_D_ = 135 ± 61 μm). To elucidate the structural basis of these specific recognitions, we determined the crystal structure of PHF20L1 Tudor1 in complex with the H3K36me1 peptide, and also solved the peptide-free structure of PHF20L1 Tudor2, which, to our knowledge, reports the first X-ray crystal structure of the PHF20L1 Tudor2 domain. Structural comparison across Tudor family readers for H3K36me and H4K20me further indicates that Tudor1 and Tudor2 rely on their aromatic cages and acidic residues to recognize specific methylated lysines. However, when we extended our analysis to full nucleosomes, we found that the tandem Tudor domains bind to DNA with high affinity and lose their methylation selectivity. Together, our findings provide a revised mechanistic framework that significantly advances the understanding of how PHF20L1 engages with chromatin, from a selective methyl-lysine reader on isolated peptides to a high-affinity DNA-binding module on nucleosomes.

## Results and discussion

### PHF20L1 Tudor domains exhibit distinct binding preferences and synergistic recognition of methylated H3K36

Although numerous studies have examined interactions between PHF20L1 Tudor domains and methyl-lysine marks (18, 19, 21, 22, 26), systematic profiling of their binding specificity and affinity across a broad panel of methylated histone peptides still lacks. Prior reports, based on histone peptide arrays, indicated that Tudor1 binds to H4K20me1 (26) and Tudor2 binds to H3K27me2 (22). To address this gap, we subcloned, expressed, and purified constructs encoding the PHF20L1 Tudor1, Tudor2, and tandem Tudor1–2 module, then performed fluorescence polarization (FP) assays using a histone peptide library covering H3K4, H3K9, H3K27, H3K36, H3K79, and H4K20 in unmethylated (Kme0) through trimethylated (Kme3) states (Fig. 1).

First, to validate earlier findings (26), we tested the binding of individual Tudor domains to H4K20 peptides with varying methylation states and found that Tudor1 binds to H4K20me1 and H4K20me2 with comparable affinities (*K*_D_ = 241 ± 34 μm and 338 ± 39 μm, respectively), showing a slight preference for the mono-methylated form. In contrast, Tudor2 displays a clear selectivity for H4K20me2 (*K*_D_ = 135 ± 61 μm) (Fig. 1, *B* and *C*, *I*). We next examined sequence selectivity by testing both Tudor domains against dimethylated peptides of H3K4, H3K9, H3K27, H3K36, and H3K79. Tudor1 binds to H3K36me2 with an affinity (*K*_D_ = 277 ± 69 μm) similar to that for H4K20me2, whereas Tudor2 shows no accurately measurable binding to any of the other dimethylated peptides tested (Fig. 1 *D* and *E*, I). This result contrasts with the previous report that detected Tudor2 binding to H3K27me2 using isothermal titration calorimetry (ITC) and surface plasmon resonance (SPR) (22). To confirm our FP observations, we performed ITC assays, which corroborated that Tudor2 prefers binding to H4K20me2 but not or only weakly to H3K27me2 (Fig. 1*H*). Independent replicate ITC titrations yielded comparable *K*_D_ values for the interactions with measurable binding affinities and supported the same relative binding trends (Fig. S1). Given Tudor1’s interaction with H3K36me2, we further tested its binding to other methylation states of H3K36. FP assays revealed that Tudor1 binds to H3K36me1 with the highest binding affinity (*K*_D_ = 133 ± 14 μm) among all the tested histone peptides (Fig. 1, *F* and *I*). We then evaluated the tandem Tudor1–2 module and found that it substantially enhances binding to H3K36me1 and H3K36me2 (*K*_D_ = 60 ± 6 μm and 88 ± 9 μm, respectively) compared with Tudor1 alone. In contrast, only modest affinity changes were observed for H4K20me1 and H4K20me2 (*K*_D_ = 210 ± 38 μm and 176 ± 24 μm, respectively) (Fig. 1, *G* and *I*). Taken together, these data indicate distinct binding preferences for the two Tudor domains: Tudor1 recognizes both mono- and di-methylated H3K36 and H4K20, with a slight preference for H3K36me1, whereas Tudor2 is highly selective for H4K20me2. In the context of the PHF20L1 tandem Tudor module, Tudor1 primarily mediates H3K36me1 recognition, while Tudor2 interacts with H4K20me2, and their tandem arrangement cooperatively strengthens the binding to methylated H3K36.

### Structural basis for the recognition of methylated histone by PHF20L1 Tudor domains

**Figure 2:**
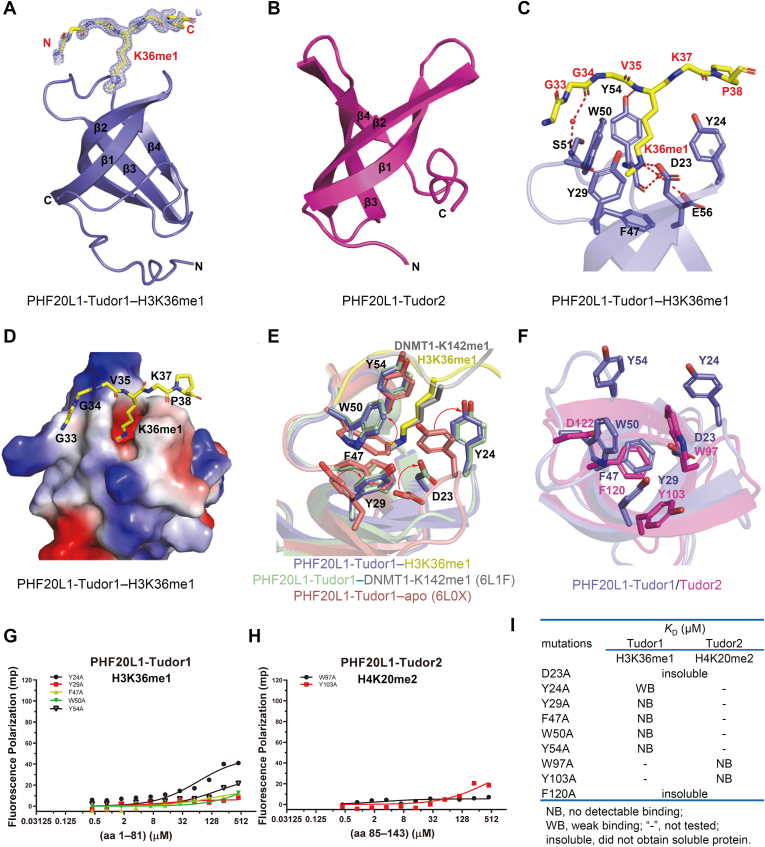
**Crystal structures of PHF20L1 Tudor1–H3K36me1 complex and apo PHF20L1 Tudor2.***A*, overall structure of PHF20L1 Tudor1 bound to H3K36me1. The protein is shown in cartoon representation in *slate* and the H3K36me1 peptide shown as stick in *yellow* with ${2F}_{o}-F_{c}$ electron density map for the peptide contoured at 1*σ* level. *B*, overall structure of PHF20L1 Tudor2 in the ligand-free (apo) state, colored in *lightmagenta*. *C*, detailed interactions between Tudor1 and H3K36me1. Amino acid residues of PHF20L1 Tudor1 involved in the H3K36me1 interaction are shown as sticks. Key hydrogen bonds are indicated by *reddashed lines* and water molecules by *red spheres*. *D*, electrostatic potential surface (*red*, negative; *blue*, positive) of Tudor1 in complex with H3K36me1, with the peptide shown as sticks. The side chains of V35 and K37 of histone H3 are omitted due to insufficient electron density. *E*, superposition of the Tudor1–H3K36me1 complex (in *slate* and *yellow*) with apo Tudor1 (PDB: 6L0X, in *salmon*) and Tudor1–DNMT1-K142me1 (PDB: 6L1F, in *pale green* and *gray*). Residues exhibiting conformational changes are marked with *red arrows*. *F*, superposition of Tudor1 (in *slate*) and Tudor2 (in *light magenta*). Potential methyl-lysine binding pocket residues are shown as sticks. *G*, FP-binding curves for Tudor1 mutants with the H3K36me1 peptide. *H*, FP-binding curves for Tudor2 mutants with the H4K20me2 peptide. *I*, summary of FP-derived binding affinities (*K*_D_) for representative PHF20L1 Tudor mutants toward the indicated histone peptides, based on (*G*) and (*H*). *K*_D_ values were obtained from duplicate measurements and fitted to a one-site binding model.

To elucidate the structural basis for selective recognition by PHF20L1 Tudor domains, we attempted to co-crystallize both the individual and tandem Tudor domains with their methylated histone ligands and determined the crystal structures of Tudor1 (residues 1–80) in complex with H3K36me1 and Tudor2 (residues 85–143) in the ligand-free (apo) state (Fig. 2 and Table 1).

**Table 1:** Data collection and refinement statistics

Complex	PHF20L1 (1–80)–H3K36me1	PHF20L1 (85–143)
PDB code	21EE	21ED
Data collection		
Space group	P2_1_2_1_2_1_	P4_1_2_1_2
Cell dimensions		
*a*, *b*, *c* (Å)	39.0, 42.5, 47.4	55.9, 55.9, 93.2
*α*, *β*, *γ* (◦)	90, 90, 90	90, 90, 90
Resolution (Å)	24.56–1.34 (1.36–1.34)	47.9–2.31 (2.39–2.31)
Measured reflections	34026 (1652)	12344 (1205)
Unique reflections	18210 (868)	6920 (652)
*R*_merge_	0.030 (0.306)	0.024 (0.260)
*I/σI*	11.6 (2.7)	13.5 (2.4)
CC_1/2_	0.998 (0.518)	0.999 (0.974)
Completeness (%)	99.9 (99.8)	100.0 (100.0)
Redundancy	1.9 (1.9)	1.8 (1.8)
Model refinement		
Resolution (Å)	24.55–1.34 (1.39–1.34)	36.36–2.31 (2.39–2.31)
*R*_*work*_/*R*_*free*_ (%)	18.9/21.3	25.7/28.4
No. of atoms/average *B*-factors (Å^2^)	717/22.3	451/66.8
Protein	647/21.3	448/66.9
Ligand	10/31.0	
Water	60/30.2	3/52.2
Root mean square deviation		
Bond lengths (Å)	0.010	0.010
Bond angles (°)	1.2	2.0
Ramachandran plot residues (%)		
Favored	100.0	100.0
Additional allowed	0.0	0.0
Disallowed	0.0	0.0

Values in parentheses are for the highest resolution shell.

Both PHF20L1 Tudor1 and Tudor2 adopt a canonical β-barrel fold consisting of four antiparallel β-strands (Fig. 2, *A* and *B*). In the Tudor1–H3K36me1 complex, residues 33 to 38 of histone H3 (sequence GGVKKP) are resolved with sufficient density except for the side chains of V35 and K37 (Fig. 2*A*). The side chain of K36me1 inserts into an aromatic cage formed by Y24, Y29, F47, W50, and Y54, engaging in van der Waals and cation–π interactions with these residues (Fig. 2, *C* and *D*). The ε-nitrogen of K36me1 donates a hydrogen bond to D23, while its backbone amide forms a hydrogen bond with the hydroxyl group of Y54. Furthermore, a water-mediated hydrogen-bond network connects the ε-nitrogen of K36me1 to the side chain of E56 and the backbone of Y54 (Fig. 2*C*). Together, these specific interactions underlie the selectivity of PHF20L1 Tudor1 for mono- and di-methylated lysine. Additionally, the backbone carbonyl of G34 hydrogen-bonds with the backbone amide of S51, which may help stabilize the N-terminal segment of the peptide (Fig. 2*C*). Beyond these contacts, no other substantial interactions are observed, consistent with the relatively weak affinity measured by FP (Fig. 1, *F* and *I*). The absence of peptide side-chain-mediated interactions apart from the methylated lysine also aligns with the lack of pronounced selectivity among the tested di-methylated peptides (Fig. 1, *D* and *I*).

Superposition of this complex with the apo structure of Tudor1 (PDB: 6L0X) reveals local conformational adjustments in D23, Y24, Y29, W50, and Y54 upon ligand binding, a pattern similarly observed in the Tudor1–DNMT1 K142me1 complex (PDB: 6L1F) (Fig. 2*E*) (27). In the apo structure of Tudor2, a corresponding aromatic cage is formed by W97, Y103, and F120 (Fig. 2*F*). Notably, an aspartate residue, D122, occupies a position analogous to W50 in Tudor1 and contributes to shaping the methyl-lysine binding pocket (Fig. 2*F*).

To validate the functional importance of the residues identified in the Tudor1–H3K36me1 complex and the predicted cage of Tudor2, we introduced point mutations into PHF20L1 Tudor1 and Tudor2 and assessed their binding. Substitution of any single aromatic cage residue in Tudor1 (Y24A, Y29A, F47A, W50A, or Y54A) markedly reduces or abolishes binding to H3K36me1 (Fig. 2, *G* and *I*). Similarly, the W97A and Y103A mutations in Tudor2 eliminate binding to H4K20me2 (Fig. 2, *H* and *I*). Mutations at D23 in Tudor1 and F120 in Tudor2 result in protein insolubility, indicating that these residues are essential for structural integrity in addition to their roles in peptide recognition (Fig. 2*I*).

To further explore the structural basis for the enhanced H3K36me1 binding by the tandem Tudor1–2 module, we performed AlphaFold-based modeling of the PHF20L1 Tudor1–2 module in complex with the H3K36me1 peptide (residues 33–44), corresponding exactly to the peptide sequence used in our FP binding assays (28). In the predicted model, the H3K36me1 peptide is positioned within the aromatic cage of Tudor1, whereas no direct interaction between H3K36me1 and Tudor2 is observed (Fig. S2). This prediction is consistent with our binding data and supports the conclusion that Tudor1 is the primary domain responsible for H3K36me1 recognition. Therefore, the enhanced affinity observed for the tandem Tudor1–2 module is unlikely to result from simultaneous engagement of H3K36me1 by both Tudor domains. Instead, Tudor2 may contribute indirectly, for example, by stabilizing the conformation of Tudor1, influencing the relative orientation or local environment of the tandem module, or reducing conformational flexibility *via* the inter-domain linker. Although the precise mechanism remains unclear, a similar phenomenon has been observed for the tandem Tudor domains of PHF20, which show enhanced binding to p53K382me2 compared to the isolated Tudor1 domain, despite Tudor1 alone exhibiting no detectable binding (26). Further investigation will be required to fully elucidate how Tudor2 potentiates H3K36me1 binding within the tandem module of PHF20L1.

### Comparison with other H3K36me and H4K20me reader Tudor domains reveals the structural basis for methyl-lysine selectivity

**Figure 3:**
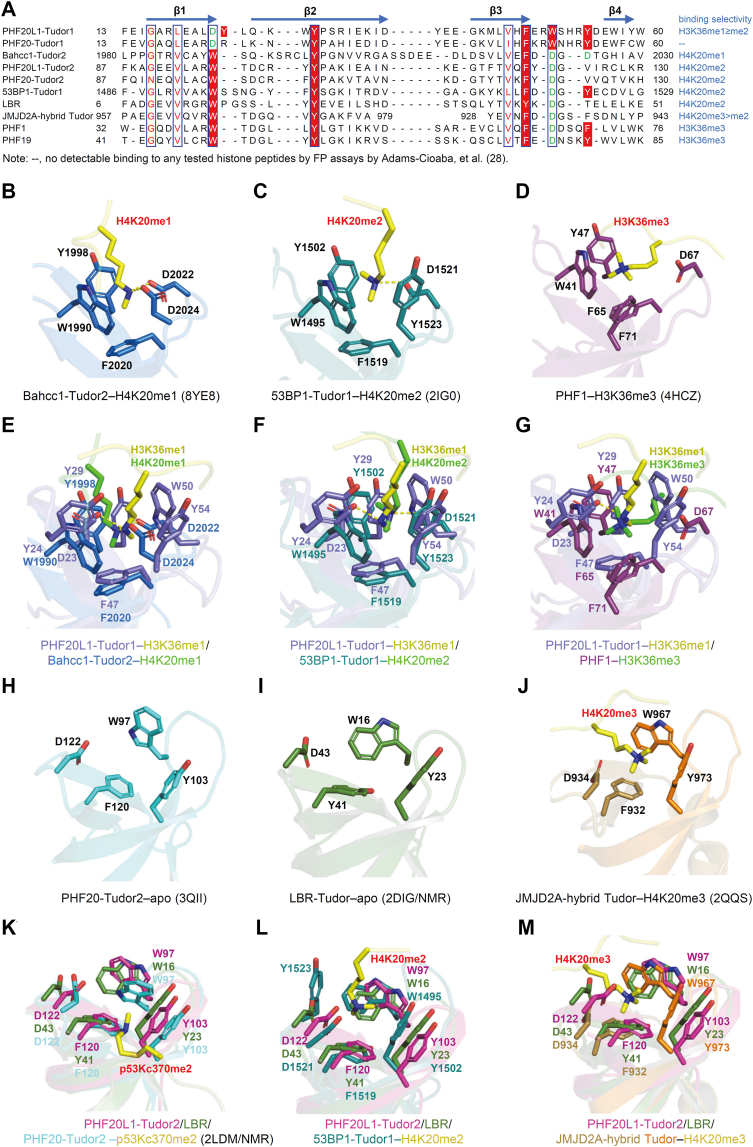
**Structural comparison of selected H3K36me and H4K20me reader Tudor domains.***A*, structural-based sequence alignment of Tudor domains from PHF20L1 (Tudor1 and Tudor2), PHF20 (Tudor1 and Tudor2), murine Bahcc1 (Tudor2), 53BP1 (Tudor1), LBR, JMJD2A (hybrid Tudor), PHF1, and PHF19. Aromatic-cage residues are highlighted in *red* background and the binding associated Asp residue in *green*. Secondary structural elements of PHF20L1 Tudor1 are indicated above the sequence with β-strands indicated by *arrows*. Binding selectivity for different methyl-lysine states is annotated to the *right*. *B*, tudor2 domain of murine Bahcc1 in complex with histone H4K20me1 peptide (PDB: 8YE8). *C*, Tudor1 domain of 53BP1 in complex with histone H4K20me2 peptide (PDB: 2IG0). (*D*) Tudor domain of PHF1 in complex with histone H3K36me3 peptide (PDB: 4HCZ). *E–G*, Superpositions with the PHF20L1 Tudor1–H3K36me1 complex (protein in *slate*, peptide in *yellow*), with murine Bahcc1 Tudor2–H4K20me1 (in *brightblue* and *green*, *E*), with 53BP1 Tudor1–H4K20me2 (in *deep teal* and *green*, *F*), with PHF1–H3K36me3 (in *violet-purple* and *green*, *G*), respectively. For clarity, only pocket-forming residues and methyl-lysine are shown. *H*, Tudor2 domain of PHF20 in the ligand-free state (PDB: 3QII). *I*, Tudor domain of LBR in the ligand-free state (PDB: 2DIG; determined by NMR). *J*, hybrid Tudor domain of JMJD2A in complex with H4K20me3 peptide (PDB: 2QQS). *K–M*, superposition of PHF20L1-Tudor2 (in *light magenta*) and LBR (in *forestgreen*), with the complex structures of PHF20 Tudor2–p53K_C_370me2 (in *cyan* and *yellow*, *K*), with the complex structures of 53BP1 Tudor1–H4K20me2 (in *deep teal* and *yellow*, *L*), with the complex structures of JMJD2A hybrid Tudor–H4K20me3 (in *brown*–*orange* and *yellow*, *M*), respectively. For clarity, only pocket-forming residues and methyl-lysine are shown.

Structure-based sequence alignment of Tudor domains with distinct binding specificities for H3K36me and H4K20me, including those from PHF20 (26, 29), murine Bahcc1 (30), 53BP1 (31), LBR (32), JMJD2A (33), PHF1 (34, 35, 36, 37), and PHF19 (35, 36, 37, 38, 39), reveals that methyl-lysine recognition is mediated by a conserved aromatic-cage formed by 3 to 5 aromatic residues, often accompanied by one or two aspartate residues (Fig. 3*A*). Notably, PHF20L1 displays a distinctive residue swap relative to other Tudor domains: D23 in PHF20L1 occupies the position typically held by the first aromatic residue (tryptophan) in other Tudors, whereas W50 in PHF20L1 substitutes the aspartate found in equivalent positions in the aligned structures. This swap is conserved in PHF20, a close homolog of PHF20L1 (Fig. 3*A*). Furthermore, PHF20L1 contains a unique aromatic residue, Y24, which also contributes to forming the methyl-lysine binding pocket. The corresponding residue in PHF20 is a positively charged residue, R24 (Fig. 3*A*). This structural divergence provides a plausible explanation for the lack of detectable binding of PHF20-Tudor1 to any tested histone peptide in FP assays (29).

Detailed structural comparisons indicate that binding selectivity is governed not only by the composition of the aromatic cage but also by the presence and spatial arrangement of acidic side chains within the pocket. In PHF20L1 Tudor1, the carboxylate side chain of D23 projects into the hydrophobic cavity and hydrogen-bonds with the remaining proton on the ε-amino group of mono- or di-methylated lysine (Kme1/2), supporting recognition of both methylation states (Fig. 2*C*). By contrast, the Bahcc1 Tudor2 pocket contains two aspartate residues (D2022 and D2024), which form two hydrogen bonds with the two protons of the ε-amino group of Kme1, and this arrangement underlies Bahcc1’s strong preference for mono-over di-methylated lysine (Fig. 3, *B* and *E*) (30). The pocket of 53BP1 Tudor1 resembles that of PHF20L1, with a single aspartate (D1521) hydrogen-bonding to the amino proton of Kme2 (Fig. 3*C*) (31). However, 53BP1 favors Kme2 over Kme1, whereas PHF20L1 Tudor1 binds both with comparable affinity, with a little preference for Kme1. Careful structural comparison suggests that the PHF20L1 Tudor1 pocket is formed by five aromatic residues, compared to four in 53BP1, which can provide more extensive van der Waals and cation–π interactions with Kme1 (Fig. 3*F*). Furthermore, the water-mediated hydrogen-bond network linking the ε-nitrogen of Kme1 to the side chain of E56 and the backbone of Y54 in PHF20L1 Tudor1 likely reinforces the interaction (Fig. 2*C*). In the case of PHF1, a more open pocket readily accommodates the tri-methylated lysine side chain (Fig. 3*D*) (34). Although PHF1 also contains an aspartate (D67) in its pocket, the methylated lysine side chain inserts from the side, positioning its amino group too far from D67 to form a hydrogen bond (Fig. 3*G*). This geometry is another key determinant of PHF1’s specificity for tri-methylated lysine over lower methylation states. Beyond differences in the binding pocket itself, residues flanking the methylated lysine also contribute to specific interactions in Bahcc1 (30), 53BP1 (31), and PHF1 (34, 35), underpinning sequence-specific recognition by these Tudor domains. This contrasts with PHF20L1 Tudor1, which shows little sequence-specific interaction outside the methyl-lysine site.

Similar to PHF20L1 Tudor2, 53BP1 Tudor1 (31), PHF20 Tudor2 (26, 29), and the LBR Tudor domain (32) have been reported to prefer H4K20me2. Structure-based sequence alignment and comparison indicate that the methyl-lysine-binding pockets of PHF20L1 Tudor2, PHF20 Tudor2, and the LBR Tudor are highly conserved, each comprising an aromatic cage of three aromatic residues and a conserved aspartate. In contrast, the 53BP1 Tudor1 pocket contains an additional tyrosine (Y1523) (Figs. 2*F* and 3, *A*, *C*, *H,* and *I*). In addition to the crystal structure of the 53BP1–H4K20me2 complex, an NMR structure of a fusion protein with a p53K_C_370me2 peptide appended to the C terminus of PHF20 Tudor2 has also been reported (26). Moreover, the hybrid Tudor domain of JMJD2A, a reader of H4K20me3, has been solved in complex with its ligand (Fig. 3*J*) (33). Superposition of the apo structures of PHF20L1 Tudor2 and LBR Tudor onto these complex structures suggests that the methylated lysine side chain could insert into the pocket from one of three possible directions (Fig. 3, *K–M*). The orientation observed in complexes with PHF20 and 53BP1 is likely similar for PHF20L1 Tudor2 and LBR Tudor (Fig. 3, *K* and *L*). Notably, however, unless conformational adjustments occur, the side chain of D122 in PHF20L1 Tudor2 would clash with the methylated lysine if it adopted the insertion geometry observed for H4K20me3 in JMJD2A (Fig. 3*M*). Given the high similarity between the pockets of PHF20L1 Tudor2, PHF20 Tudor2, and 53BP1 Tudor1, the selectivity rules established for these domains likely apply to PHF20L1 Tudor2 as well. In summary, the aromatic cage provides a hydrophobic and cation–π environment for methyl-lysine encapsulation, while the presence, number, and orientation of aspartate residues within the pocket modulate hydrogen-bonding potential, thereby fine-tuning specificity across Kme1, Kme2, and Kme3 states.

### Nucleosome binding assays reveal that the tandem Tudor domains of PHF20L1 bind to DNA with high affinity and lose methylation selectivity

To investigate the binding properties of PHF20L1 Tudor domains in a physiologically relevant chromatin context, we performed biolayer interferometry (BLI) assays using reconstituted nucleosome core particles (NCPs) carrying methylated lysine analogue (MLA) H3K_C_36me1, H4K_C_20me2, or both modifications, as well as free 601 DNA (Figs. 4, S3–S14, and Table 2).

**Figure 4:**
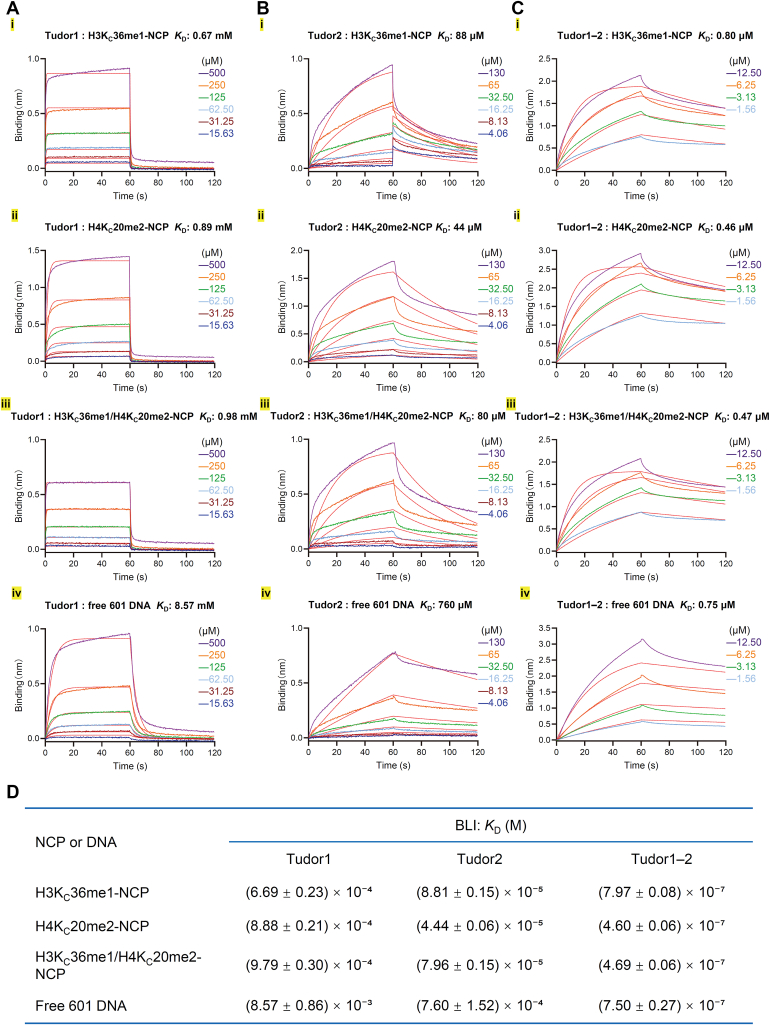

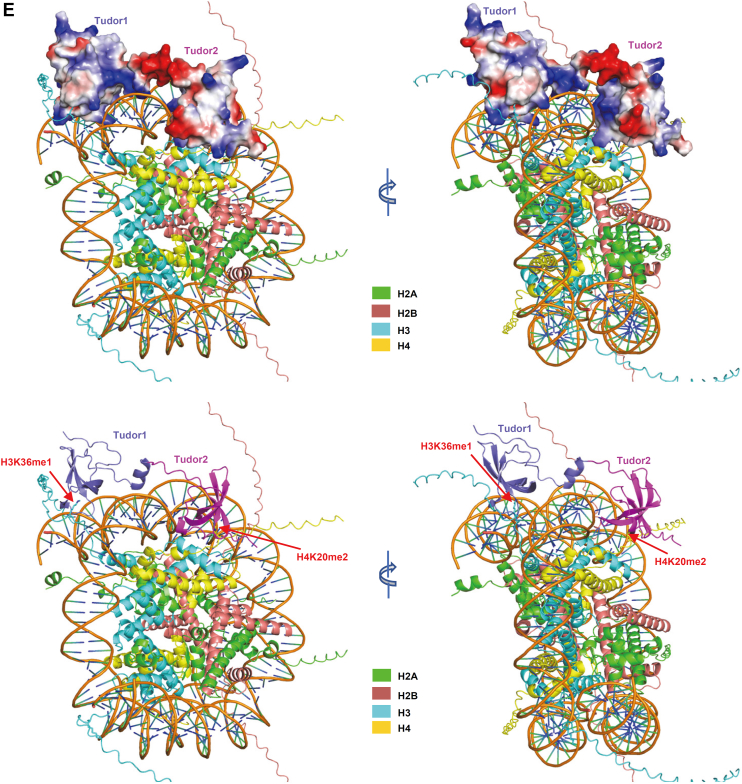
**Tandem Tudor domains of PHF20L1 bind to DNA with high affinity without methylation selectivity.***A*, BLI binding curves for PHF20L1 Tudor1 binding to biotinylated 601 DNA-bearing NCPs containing H3K_C_36me1 (i), H4K_C_20me2 (ii), or both modifications (iii), as well as to biotinylated free 601 DNA (iv). *B*, BLI binding curves for PHF20L1 Tudor2 binding to the same set of NCPs (i–iii) and free 601 DNA (iv). *C*, BLI binding curves for PHF20L1 Tudor1–2 binding to the same set of NCPs (i–iii) and free 601 DNA (iv). *D*, Summary of binding affinities (*K*_D_) for the different PHF20L1 Tudor domains interacting with biotinylated 601 DNA-containing MLA NCPs (or free 601 DNA) as determined by BLI. All assays were performed using an Octet-R2 instrument (Sartorius) at 30 °C, with biotinylated MLA NCPs immobilized on streptavidin biosensors and incubated with increasing concentrations of the respective PHF20L1 Tudor domains. Data shown are representative of two independent experiments. *E*, structural model of the tandem Tudor domains in complex with H3K36me1/H4K20me2-containing nucleosome, generated using AlphaFold 3 and shown in electrostatic potential surface representation (*upper*; *red*, negative; *blue*, positive) and cartoon representation (*lower*).

**Table 2:** Summary of binding parameters for the different PHF20L1 Tudor domains interacting with biotinylated 601 DNA-containing MLA NCPs (or free 601 DNA) as determined by BLI

NCP or DNA	Protein	*K*_D_ (M)	*k*_on_ (1/Ms)	*k*_off_ (1/s)	*R*^2^
H3K_C_36me1-NCP	Tudor1	(6.69 ± 0.23) × 10^−4^	(3.80 ± 0.09) × 10^3^	2.54 ± 0.06	0.99
	Tudor2	(8.81 ± 0.15) × 10^−5^	(2.03 ± 0.03) × 10^2^	(1.78 ± 0.01) × 10^−2^	0.96
	Tudor1–2	(7.97 ± 0.08) × 10^−7^	(6.43 ± 0.03) × 10^3^	(5.12 ± 0.04) × 10^−3^	0.98
H4K_C_20me2-NCP	Tudor1	(8.88 ± 0.21) × 10^−4^	(4.72 ± 0.09) × 10^2^	(4.19 ± 0.05) × 10^−^	0.98
	Tudor2	(4.44 ± 0.06) × 10^−5^	(3.27 ± 0.04) × 10^2^	(1.45 ± 0.01) × 10^−2^	0.96
	Tudor1–2	(4.60 ± 0.06) × 10^−7^	(8.45 ± 0.04) × 10^3^	(3.88 ± 0.04) × 10^−3^	0.97
H3K_C_36me1/H4K_C_20me2-NCP	Tudor1	(9.79 ± 0.30) × 10^−4^	(4.22 ± 0.09) × 10^3^	4.13 ± 0.09	0.99
	Tudor2	(7.96 ± 0.15) × 10^−5^	(2.81 ± 0.05) × 10^2^	(2.24 ± 0.02) × 10^−2^	0.94
	Tudor1–2	(4.69 ± 0.06) × 10^−7^	(7.79 ± 0.04) × 10^3^	(3.65 ± 0.04) × 10^−3^	0.97
Free 601 DNA	Tudor1	(8.57 ± 0.86) × 10^−3^	(2.43 ± 0.24) × 10	(2.09 ± 0.02) × 10^−1^	0.99
	Tudor2	(7.60 ± 1.52) × 10^−4^	8.19 ± 1.64	(6.23 ± 0.06) × 10^−3^	0.98
	Tudor1–2	(7.50 ± 0.27) × 10^−7^	(2.85 ± 0.02) × 10^3^	(2.14 ± 0.07) × 10^−3^	0.94

The BLI data revealed that individual Tudor domains show weak and non-selective nucleosome binding. Tudor1 binds to all three types of methylated nucleosomes with dissociation constants (*K*_D_) ranging from ∼6.7 × 10^−4^ M to ∼9.8 × 10^−4^ M, whereas Tudor2 exhibits *K*_D_ values of ∼4.4 × 10^−5^ M to ∼8.8 × 10^−5^ M (Fig. 4, *A* and *B*, *D*). No significant preference for any specific methylation mark was observed for either domain. Both Tudor1 and Tudor2 bind to free 601 DNA even more weakly (*K*_D_ ≈ 8.6 × 10^−3^ M and 7.6 × 10^−4^ M, respectively, Fig. 4, *A* and *B*, *D*), suggesting that the histone components contribute modestly to nucleosome recognition. Nevertheless, the overall affinities remain in the weak (μm to mM) range. In striking contrast, the tandem Tudor1–2 module binds to nucleosomes and free DNA with similarly high affinity and without methylation selectivity (Fig. 4, *C* and *D*). The tandem module displays sub-micromolar *K*_D_ values of approximately 4.6 × 10^−7^ M to 8.0 × 10^−7^ M for all nucleosome substrates, which are statistically indistinguishable from its affinity for free 601 DNA (*K*_D_ ≈ 7.5 × 10^−7^ M).

Kinetic analysis revealed that the tandem module has markedly increased association rate constants (*k*_on_) and decreased dissociation rate constants (*k*_off_) compared to the individual domains. For example, binding to H3K36me1 nucleosomes gives *k*_on_ values of 3.80 × 10^3^ M^−1^s^−1^ (Tudor1), 2.03 × 10^2^ M^−1^s^−1^ (Tudor2), and 6.43 × 10^3^ M^−1^s^−1^ (tandem); and *k*_off_ values of 2.54 s^−1^, 1.78 × 10^−2^ s^−1^, and 5.12 × 10^−3^ s^−1^, respectively (Table 2). These kinetic parameters are characteristic of cooperative binding (40). Importantly, the tandem module shows no detectable preference among differently methylated nucleosomes, and its binding parameters for nucleosomes and free DNA were essentially identical. These results strongly indicate that, at the nucleosome level, the high-affinity interaction of the tandem Tudor domains is primarily driven by the DNA backbone, and that the methylation-specific recognition observed with isolated histone peptides is either very weak or completely masked in the context of the full nucleosome. This conclusion is consistent with the structural model of the tandem Tudor domains in complex with H3K36me1/H4K20me2-nucleosome generated using AlphaFold 3 (28), which shows the Tudor domains binding to nucleosome DNA *via* a basic surface (Fig. 4*E*).

This DNA-driven binding mode contrasts with that of other tandem Tudor domains. Notably, although the tandem Tudor domains of 53BP1 also bind to DNA (41), their recruitment to DNA double-strand breaks is driven by H4K20me2 recognition (31). In contrast, PHF20L1 tandem Tudor domains engage the DNA backbone cooperatively, resulting in methylation-independent, sub-micromolar affinity.

In summary, our systematic nucleosome binding experiments revealed a previously unappreciated mode of interaction for the PHF20L1 tandem Tudor domains. Integrating the peptide- and nucleosome-level data, we propose a dual-mode model. On isolated histone peptides, Tudor1 and Tudor2 rely on their aromatic cages and acidic residues to recognize specific methylated lysines (H3K36me1 for Tudor1; H4K20me2 for Tudor2), but these interactions are of low affinity (high μM *K*_D_) and insufficient for stable chromatin association. On full nucleosomes, the tandem Tudor domains achieve sub-micromolar affinity through cooperative DNA backbone recognition, with no detectable methylation selectivity. Thus, the tandem Tudor domains may primarily function as a high-affinity DNA-binding module that anchors PHF20L1 to chromatin, while the methyl-lysine reader activity plays auxiliary or context-dependent roles, such as recognizing methylated non-histone substrates (*e.g.*, pRb, DNMT1), fine-tuning localization in DNA-restricted regions, or cooperating with the PHD finger domain. Our findings provide a revised mechanistic framework for understanding how PHF20L1 engages chromatin and raise important questions for future studies, including the precise mode of DNA recognition and the potential synergy between DNA-binding and methyl-lysine-binding activities in living cells.

## Experimental procedures

### Protein expression and purification

DNA fragments encoding PHF20L1 Tudor1 (residues 1–80), Tudor2 (residues 85–143), and the tandem Tudor1–2 construct (residues 1–280) were subcloned into a pET28-MHL vector to generate N-terminal 6 × His-TEV (tobacco etch virus)-tagged fusion protein. All plasmids were constructed using seamless assembly cloning (ABclonal Technology, RK21020) and verified by Sanger sequencing (Azenta Life Sciences).

Recombinant proteins were overexpressed in *E. coli* BL21 (DE3) Codon Plus RIL cells (TransGen, CD601). Cultures were induced at OD_600_ ≈ 0.8 with 0.25 mM IPTG (isopropyl-β-D-thiogalactoside) and grown for 24 h at 15 °C. Cells were harvested and lysed, and the clarified lysate was applied to Ni^2+^-nitrilotriacetate resin (GE Healthcare, 17526802). Bound proteins were washed and eluted under the following Ni-affinity conditions: Lysis Buffer: 20 mM Tris-HCl, pH 7.5, 250 mM NaCl, 5% glycerol, and 5 mM β-mercaptoethanol (β-ME); Wash Buffer: 20 mM Tris-HCl, pH 7.5, 1 M NaCl, and 40 mM imidazole; Elution Buffer: 20 mM Tris-HCl, pH 7.5, 250 mM NaCl, and 250 mM imidazole. The His tag was removed by TEV protease digestion, followed by a second Ni-affinity step to remove the His tag, uncleaved protein and the TEV protease. Proteins were further purified by size-exclusion chromatography (Superdex 75, GE Healthcare) in 20 mM Tris-HCl, pH 7.5, 150 mM NaCl, 1 mM DTT. Purified proteins were concentrated to 8 mg/ml for Tudor1, 15 mg/ml for Tudor2, and 10 mg/ml for Tudor1–2 using Amicon Ultra-15 Centrifugal Filters (Millipore, UFC901024) for fluorescence polarization (FP), isothermal titration calorimetry (ITC), and crystallization assays. All the mutations involved in this study were constructed using the Fast Mutagenesis System Kit (Transgene, FM111-02) according to the manufacturer’s instructions and confirmed by DNA sequencing. Mutants were overexpressed and purified as described for the wild-type proteins.

### Fluorescence polarization (FP) assay

All the peptides used for fluorescence polarization (FP), including H3K4 (residues 1–11), H3K9 (residues 1–11), H3K27 (residues 19–33), H3K36 (residues 33–41), H3K79 (residues 71–85), and H4K20 (residues 12–27) with different methylation states, were synthesized and purified by Tufts University Core Services with fluorescein-labeled C termini. Binding reactions (10 μl) contained 40 nM labeled peptide and titrated protein (low-to high-micromolar range) in 20 mM Tris-HCl, pH 7.5, 150 mM NaCl, 1 mM DTT, and 0.01% Triton X-100. All assays were performed in duplicate in 384-well plates, using the Synergy 2 microplate reader (BioTek) with an excitation wavelength of 485 nm and an emission wavelength of 528 nm. Data were corrected for the background of the free labeled peptides and fitted to the ligand binding function using GraphPad Prism 8 software to determine the *K*_D_ values.

### Isothermal titration calorimetry (ITC) assay

For the ITC measurement, concentrated proteins were diluted into the ITC buffer (20 mM Tris-HCl, pH 7.5, 150 mM NaCl), and lyophilized peptides (H3K27me2 (residues 19–33), H4K20me2 (residues 14–27), Shanghai Apeptide CO, Ltd or GLS GL Biochem (Shanghai) Ltd) were dissolved in the same buffer with pH adjusted by adding 2 M NaOH dropwise. Peptide concentrations were estimated based on their mass. All the measurements were conducted in duplicate at 25 °C, utilizing an iTC200 (MicroCal, Inc) microcalorimeter. In the cell chamber, the protein with a concentration of 100 μm was placed, and peptides with a concentration of 1 mM in the syringe were injected into the cell chamber for 20 successive injections with a spacing of 150 s. Control experiments were carried out under identical conditions to determine the heat signals that resulted from injecting peptides into the buffer. Data were fitted using the single-site binding model after subtraction of the control heat signals within the Origin software package (MicroCal, Inc).

### Protein crystallization

Purified PHF20L1 Tudor1 with a concentration of 8 mg/ml was mixed with methylated H3K36 (residues 28–42) or H4K20 (residues 14–27) histone peptide at a molar ratio 1:3 and crystallized using the sitting-drop vapor diffusion method at 18 °C by mixing 0.5 μl of the protein–peptide mixture with 0.5 μl of the reservoir solution. The complex of PHF20L1 Tudor1–H3K36me1 crystallized in a buffer containing 0.1 M HEPES sodium, pH 7.5, 1.4 M Sodium citrate tribasic dihydrate. As for the crystal of PHF20L1 Tudor2, purified PHF20L1 Tudor2 protein (15 mg/ml) was mixed with H4K20me2 peptide at a molar ratio of 1:3 and crystallized in a buffer containing 0.2 M ammonium sulfate, 0.1 M Bis-Tris, pH 6.5 and 25% w/v polyethylene glycol 3350. Before flash freezing crystals in liquid nitrogen, crystals were transferred briefly into cryoprotectant consisting of 85% reservoir solution and 15% glycerol.

### Data collection and structure determination

X-ray diffraction data were collected at 100 K on beamline BL10U2 of the Shanghai Synchrotron Radiation Facility (SSRF, China). Diffraction images were indexed, integrated, and scaled with XDS (42) and then assessed and merged with POINTLESS (43) and AIMLESS (44). Molecular replacement was performed with PHASER (45) using the known structure (PDB: 3Q1J for Tudor1–H3K36me1 complex and 3QII for Tudor2, respectively) as the search model. The initial model underwent restrained refinement with REFMAC (46), followed by automated rebuilding with ARP/wARP (47) and manual model building in COOT (48). Final refinement was conducted iteratively with REFMAC (46) and PHENIX (49). Data collection and refinement statistics are summarized in Table 1. All the structural figures were prepared in PyMOL.

### Reconstitution of methylated lysine analog**ue** (MLA) nucleosome core particles (NCPs)

Recombinant MLA NCPs were reconstituted as described previously with minor modifications (50). Briefly, histones H3K36C and H4K20C were generated by site-directed mutagenesis. Histones were expressed in *E. coli*, purified from inclusion bodies, and further purified by semi-preparative HPLC. For H3K_C_36me1 synthesis, purified H3K36C was reacted with (2-bromoethyl)methylammonium chloride under reducing conditions; for H4K_C_20me2, (2-bromoethyl)dimethylammonium chloride was used in a two-step addition to minimize side reactions. Products were confirmed by mass spectrometry. Equimolar amounts of H2A, H2B, H3 (or H3K_C_36me1), and H4 (or H4K_C_20me2) were mixed and refolded by dialysis against refolding buffer (10 mM Tris-HCl, pH 7.5, 2 M NaCl, 1 mM EDTA, and 1 mM DTT) at 4 °C. The histone octamer was purified by size-exclusion chromatography (Superdex 200, GE Healthcare). The Widom 601 DNA template was amplified with 5′-biotinylated primers and purified by anion-exchange chromatography (MonoQ, GE Healthcare). Purified octamer and 601 DNA were mixed at a 1.1:1 M ratio in high-salt buffer, then gradually dialyzed against TE buffer (10 mM Tris-HCl, pH 7.5, 1 mM EDTA, 1 mM DTT) to lower the NaCl concentration to 0.2 M.

### Biolayer interferometry (BLI) assay

For the BLI measurement, PHF20L1 Tudor domains were buffer-exchanged into PBS and serially diluted in PBST (PBS containing 0.05% Tween 20) prior to binding assays. Before each experiment, streptavidin (SA) biosensors were pre-wetted in PBST to establish a stable baseline. Biotinylated MLA NCPs containing the 601 DNA sequence were immobilized onto SA biosensors through a three-step procedure: baseline recording, ligand loading, and a second baseline recording, thereby generating NCP-immobilized sample biosensors. In parallel, blank reference biosensors (without immobilized NCPs) were prepared and subjected to the same procedure in PBST buffer only.

For kinetic measurements, both sample (with immobilized NCPs) and blank reference biosensors were simultaneously incubated with the same serial dilutions of PHF20L1 Tudor protein at increasing concentrations. Each concentration cycle consisted of three steps: a 60-s baseline acquisition, a 60-s association phase, and a 60-s dissociation phase. The responses from the reference biosensors were subtracted from those of the sample biosensors to correct for non-specific binding and bulk refractive-index effects. The resulting reference-subtracted binding curves were used to calculate the association rate constant (*k*_on_), dissociation rate constant (*k*_off_), and equilibrium dissociation constant (*K*_D_).

The same experimental and data-processing procedures were applied to BLI assays measuring the binding of PHF20L1 Tudor proteins to biotinylated free 601 DNA. All assays were performed at 30 °C with continuous shaking at 1000 rpm using an Octet-R2 system (Sartorius). Raw kinetic data were collected using Data Acquisition software (Octet BLI Discovery 13.0), and kinetic parameters were determined using Data Analysis software (Octet Analysis Studio 13.0).

## Data availability

The coordinates and structure factors of this study have been deposited in the Protein Data Bank (PDB) under accession codes 21EE for PHF20L1 Tudor1–H3K36me1 complex, and 21ED for ligand-free PHF20L1 Tudor2.

## Supporting information

This article contains supporting information.

## Conflict of interest

The authors declare that they have no conflicts of interest with the contents of this article.
