# Supplementary material for: A revised model for PHF20L1 Tudor function: DNA binding overrides methylation selectivity on nucleosomes
Source: J Biol Chem. 2026 May 21;302(7):113181. doi: 10.1016/j.jbc.2026.113181 (PMC13285377; doi:10.1016/j.jbc.2026.113181)
Supplement: Supplementary Material [file mmc1.pdf]

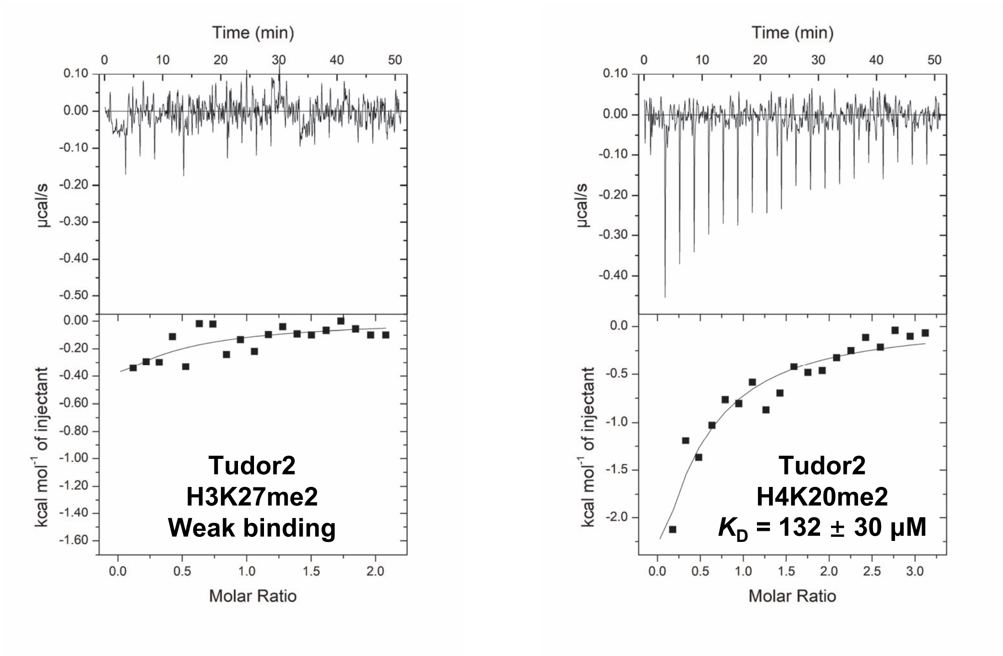

Supplementary Figure 1. Independent replicate ITC analysis of the interaction between PHF20L1 Tudor2 and H3K27me2 or H4K20me2. Representative independent ITC titrations of PHF20L1 Tudor2 with H3K27me2 (left) or H4K20me2 (right) peptide.

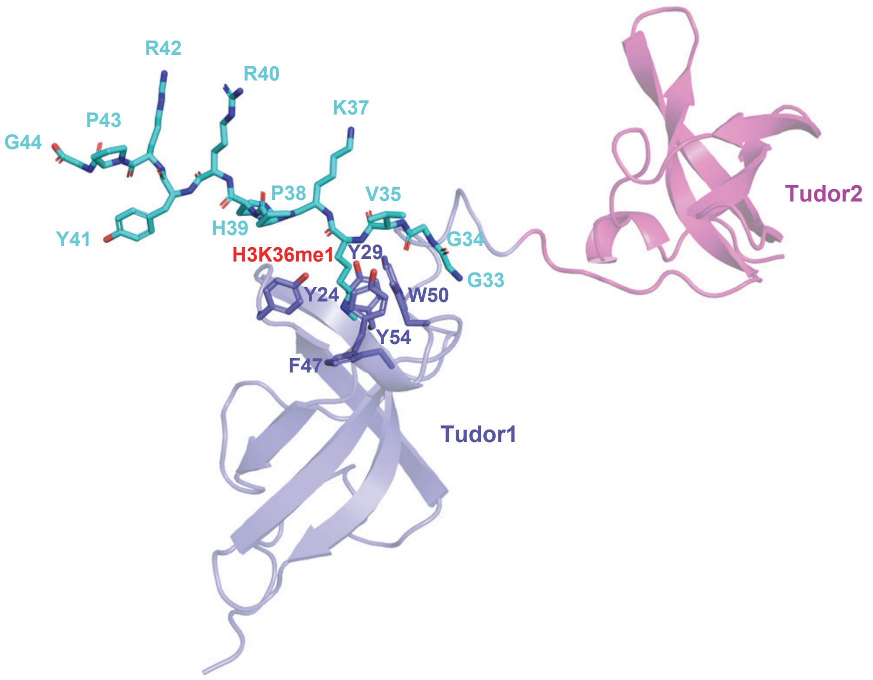

AlphaFold-PHF20L1-Tudor1–Tudor2–H3K36me1

Supplementary Figure 2. AlphaFold-based structural model of the PHF20L1 Tudor1–Tudor2 tandem module in complex with the H3K36me1 peptide. Predicted structural model of the PHF20L1 Tudor1–Tudor2 tandem module bound to the H3K36me1 peptide (residues 33–44). Tudor1 is shown in blue, Tudor2 in magenta, and the H3K36me1 peptide in cyan.

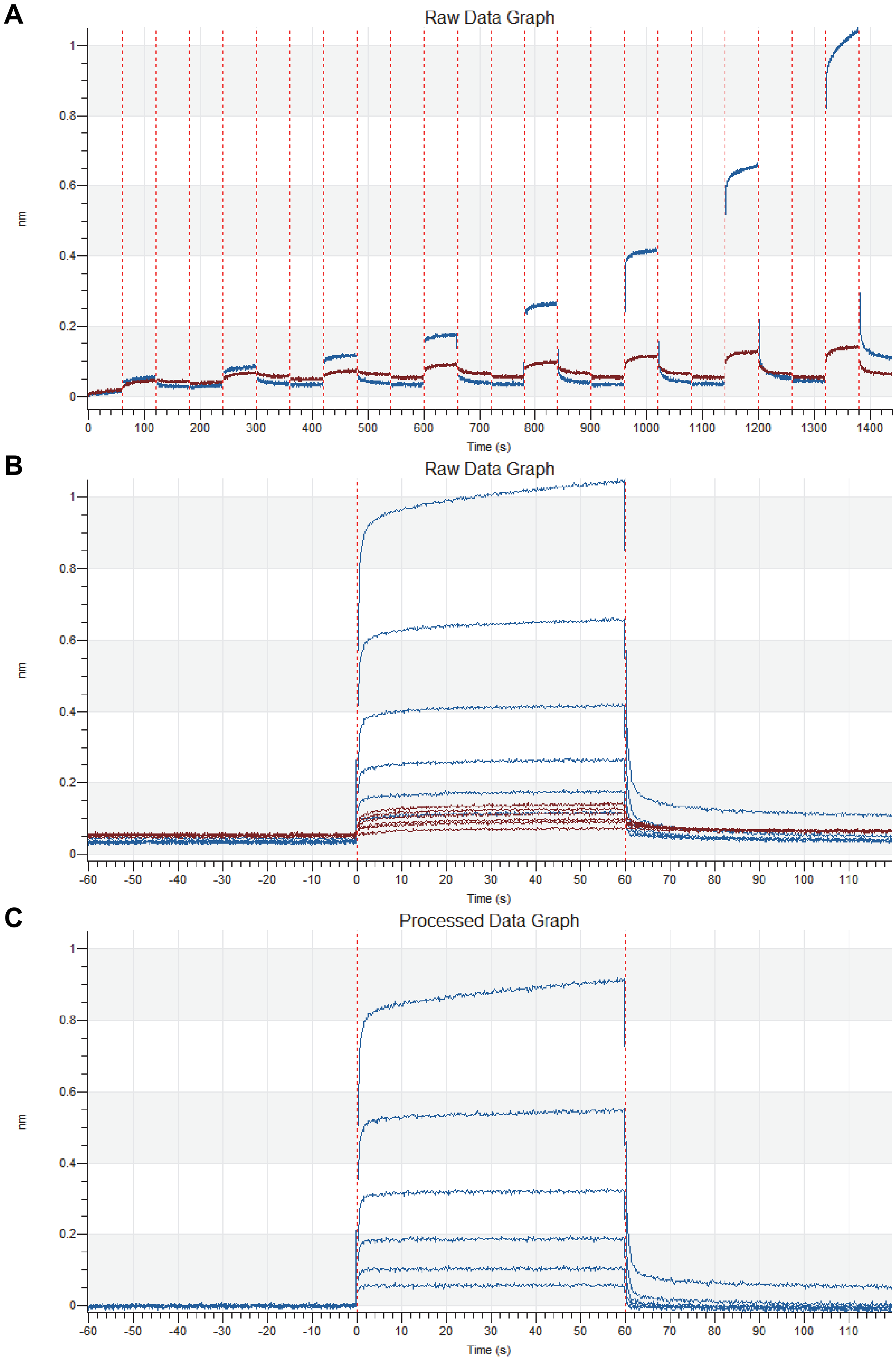

Supplementary Figure 3. BLI data processing for the interaction between H3K$_C$36me1-NCP and PHF20L1 Tudor1.

(A) Raw sensorgrams recorded using H3K$_C$36me1-NCP-immobilized biosensors (blue) and blank reference biosensors (red).

(B) Overlay of the raw sensorgrams at increasing concentrations of PHF20L1 Tudor1 prior to reference subtraction.

(C) Reference-subtracted binding curves generated by subtracting blank biosensor signals from H3K$_C$36me1-NCP-immobilized biosensor signals. These processed curves were subsequently used for kinetic fitting and calculation of the binding parameters, including K_D, k_{on}, and k_{off}.

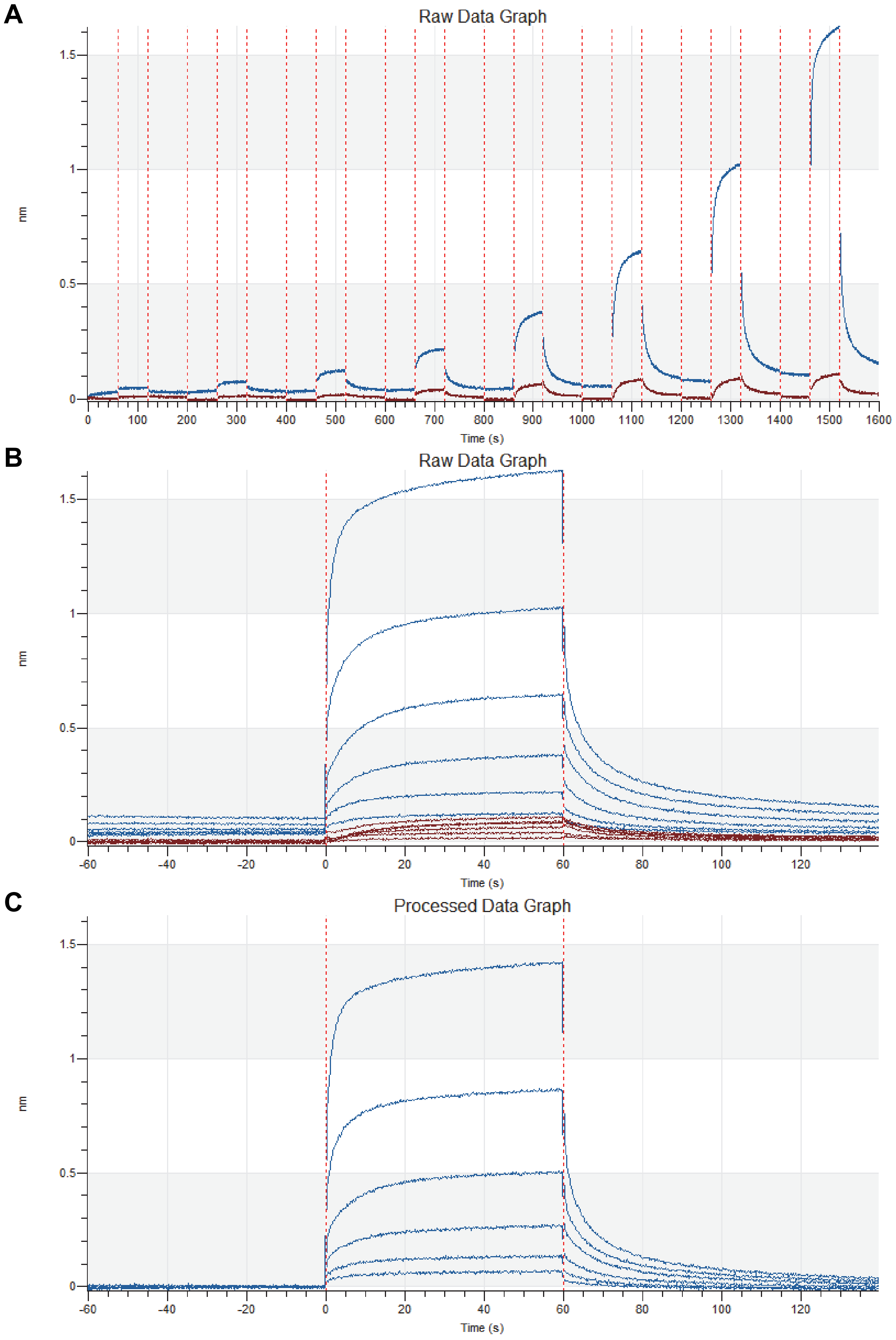

Supplementary Figure 4. BLI data processing for the interaction between H4K$_C$20me2-NCP and PHF20L1 Tudor1.

(A) Raw sensorgrams recorded using H4K$_C$20me2-NCP-immobilized biosensors (blue) and blank reference biosensors (red).

(B) Overlay of the raw sensorgrams at increasing concentrations of PHF20L1 Tudor1 prior to reference subtraction.

(C) Reference-subtracted binding curves generated by subtracting blank biosensor signals from H4K$_C$20me2-NCP-immobilized biosensor signals. These processed curves were subsequently used for kinetic fitting and calculation of the binding parameters, including K_D, k_{on}, and k_{off}.

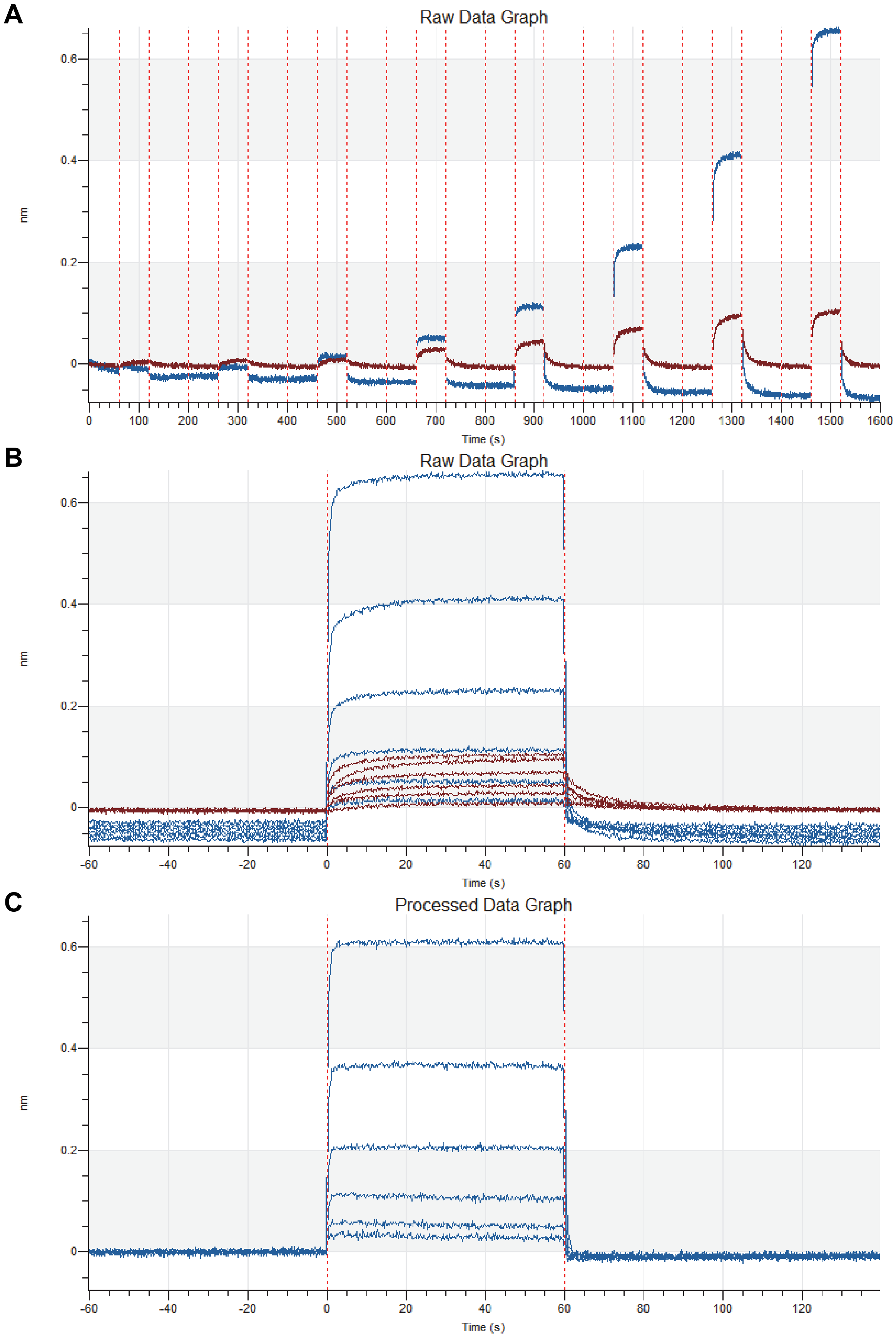

Supplementary Figure 5. BLI data processing for the interaction between H3K$_C$36me1/H4K$_C$20me2-NCP and PHF20L1 Tudor1.

(A) Raw sensorgrams recorded using H3K$_C$36me1/H4K$_C$20me2-NCP-immobilized biosensors (blue) and blank reference biosensors (red).

(B) Overlay of the raw sensorgrams at increasing concentrations of PHF20L1 Tudor1 prior to reference subtraction.

(C) Reference-subtracted binding curves generated by subtracting blank biosensor signals from H3K$_C$36me1/H4K$_C$20me2-NCP-immobilized biosensor signals. These processed curves were subsequently used for kinetic fitting and calculation of the binding parameters, including K_D, k_{on}, and k_{off}.

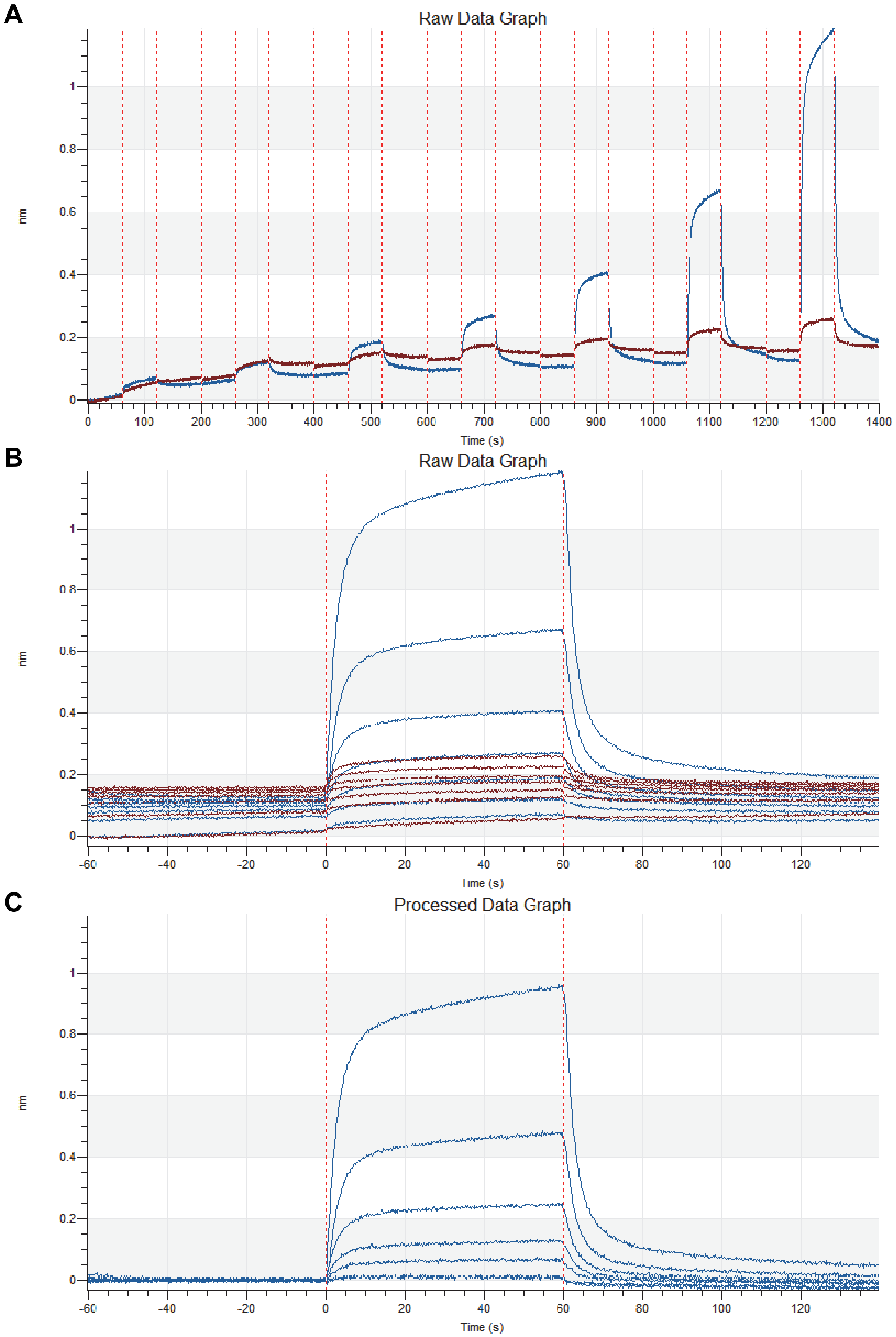

Supplementary Figure 6. BLI data processing for the interaction between free 601 DNA and PHF20L1 Tudor1.

(A) Raw sensorgrams recorded using free 601 DNA-immobilized biosensors (blue) and blank reference biosensors (red).

(B) Overlay of the raw sensorgrams at increasing concentrations of PHF20L1 Tudor1 prior to reference subtraction.

(C) Reference-subtracted binding curves generated by subtracting blank biosensor signals from free 601 DNA-immobilized biosensor signals. These processed curves were subsequently used for kinetic fitting and calculation of the binding parameters, including K_D, k_{on}, and k_{off}.

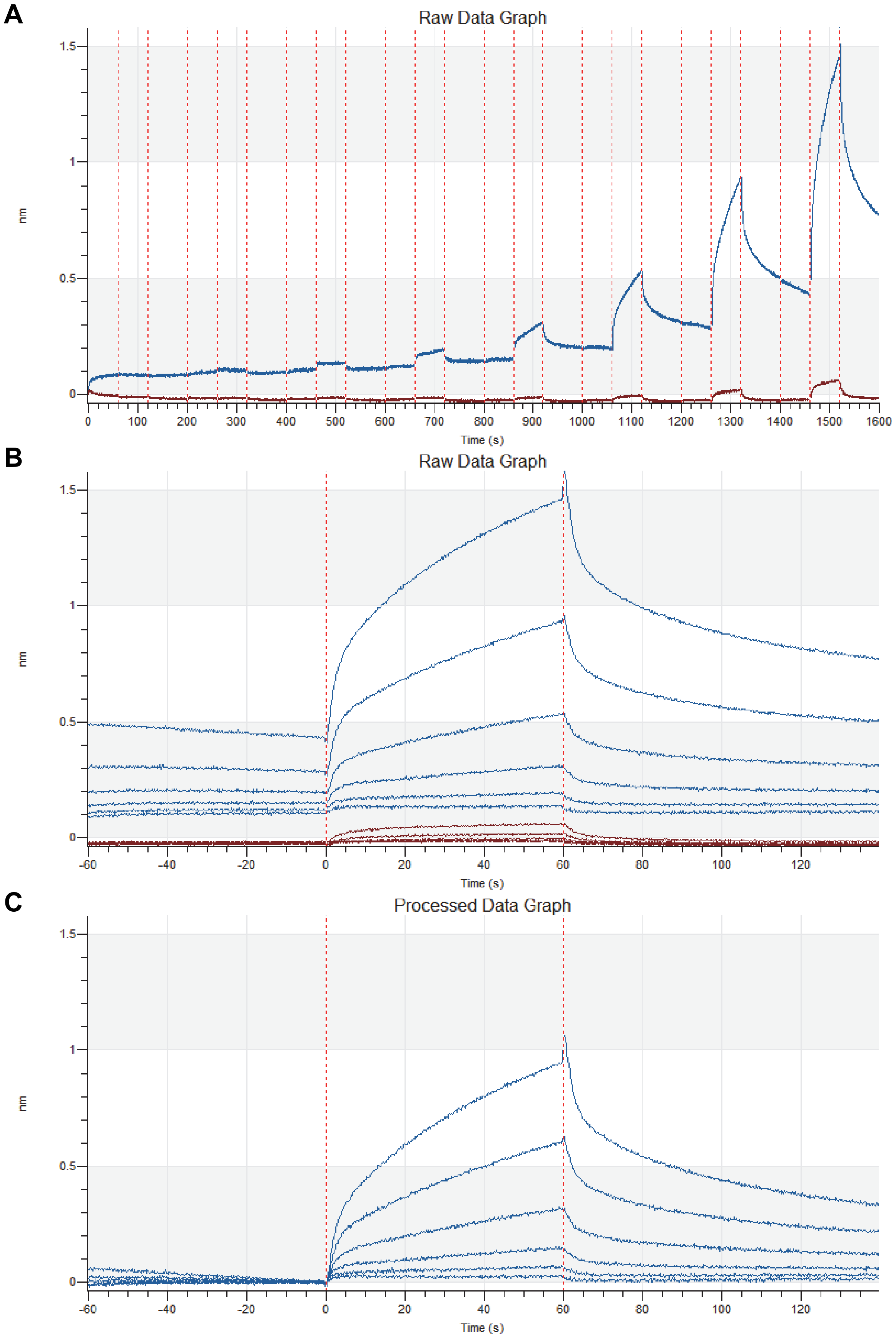

Supplementary Figure 7. BLI data processing for the interaction between H3K$_C$36me1-NCP and PHF20L1 Tudor2.

(A) Raw sensorgrams recorded using H3K$_C$36me1-NCP-immobilized biosensors (blue) and blank reference biosensors (red).

(B) Overlay of the raw sensorgrams at increasing concentrations of PHF20L1 Tudor2 prior to reference subtraction.

(C) Reference-subtracted binding curves generated by subtracting blank biosensor signals from H3K$_C$36me1-NCP-immobilized biosensor signals. These processed curves were subsequently used for kinetic fitting and calculation of the binding parameters, including K_D, k_{on}, and k_{off}.

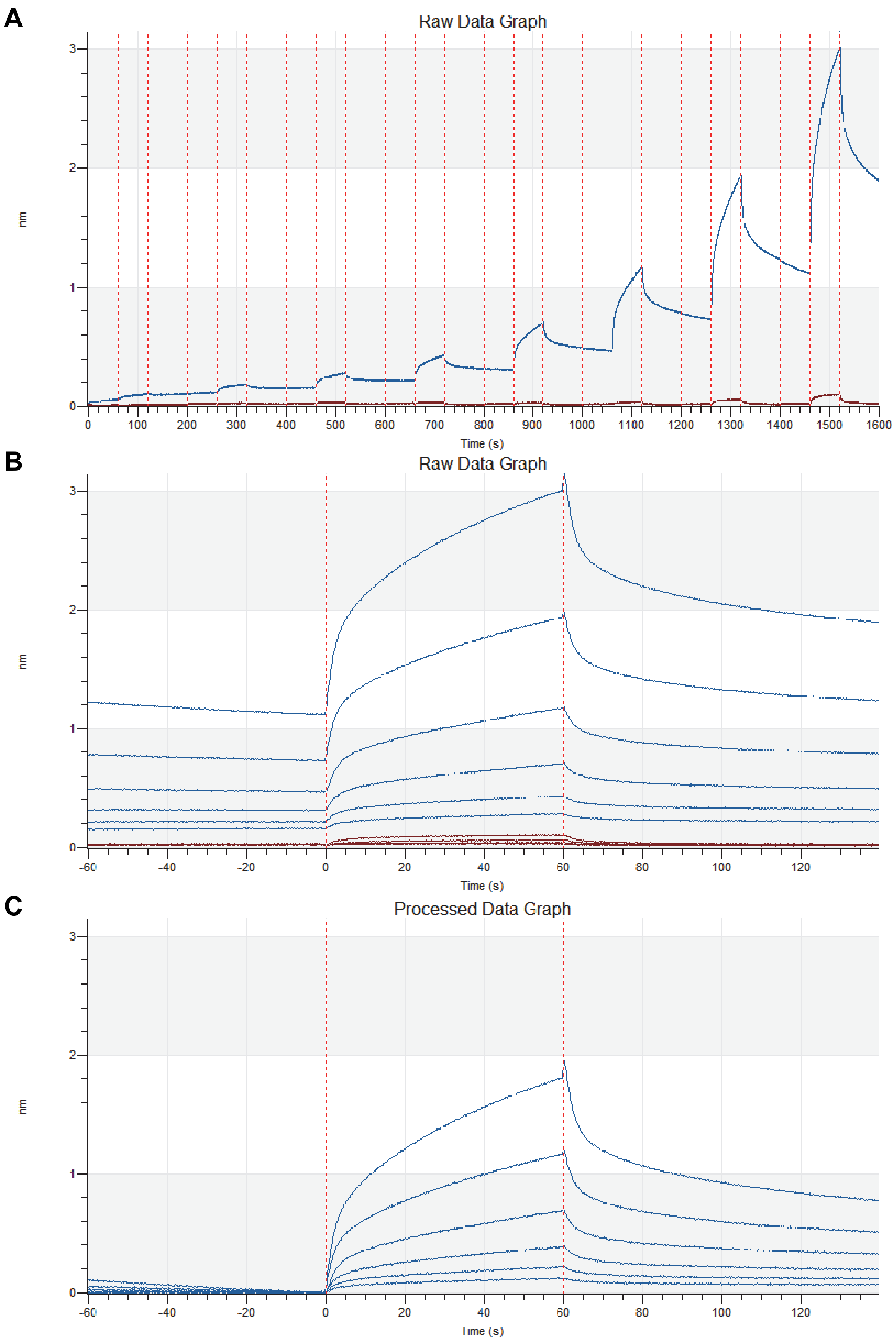

Supplementary Figure 8. BLI data processing for the interaction between H4K$_C$20me2-NCP and PHF20L1 Tudor2.

(A) Raw sensorgrams recorded using H4K$_C$20me2-NCP-immobilized biosensors (blue) and blank reference biosensors (red).

(B) Overlay of the raw sensorgrams at increasing concentrations of PHF20L1 Tudor2 prior to reference subtraction.

(C) Reference-subtracted binding curves generated by subtracting blank biosensor signals from H4K$_C$20me2-NCP-immobilized biosensor signals. These processed curves were subsequently used for kinetic fitting and calculation of the binding parameters, including K_D, k_{on}, and k_{off}.

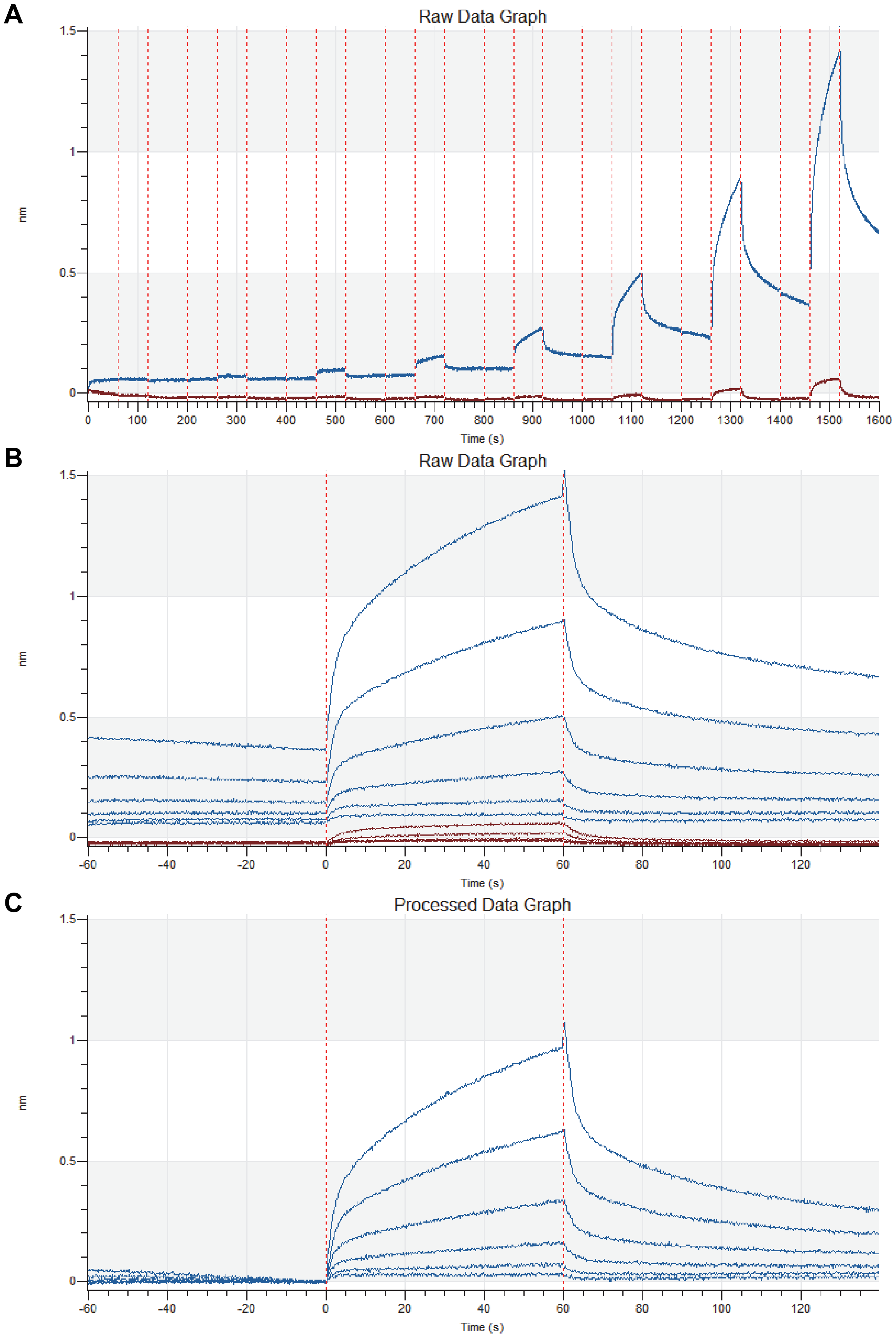

Supplementary Figure 9. BLI data processing for the interaction between H3K$_C$36me1/H4K$_C$20me2-NCP and PHF20L1 Tudor2.

(A) Raw sensorgrams recorded using H3K$_C$36me1/H4K$_C$20me2-NCP-immobilized biosensors (blue) and blank reference biosensors (red).

(B) Overlay of the raw sensorgrams at increasing concentrations of PHF20L1 Tudor2 prior to reference subtraction.

(C) Reference-subtracted binding curves generated by subtracting blank biosensor signals from H3K$_C$36me1/H4K$_C$20me2-NCP-immobilized biosensor signals. These processed curves were subsequently used for kinetic fitting and calculation of the binding parameters, including K_D, k_{on}, and k_{off}.

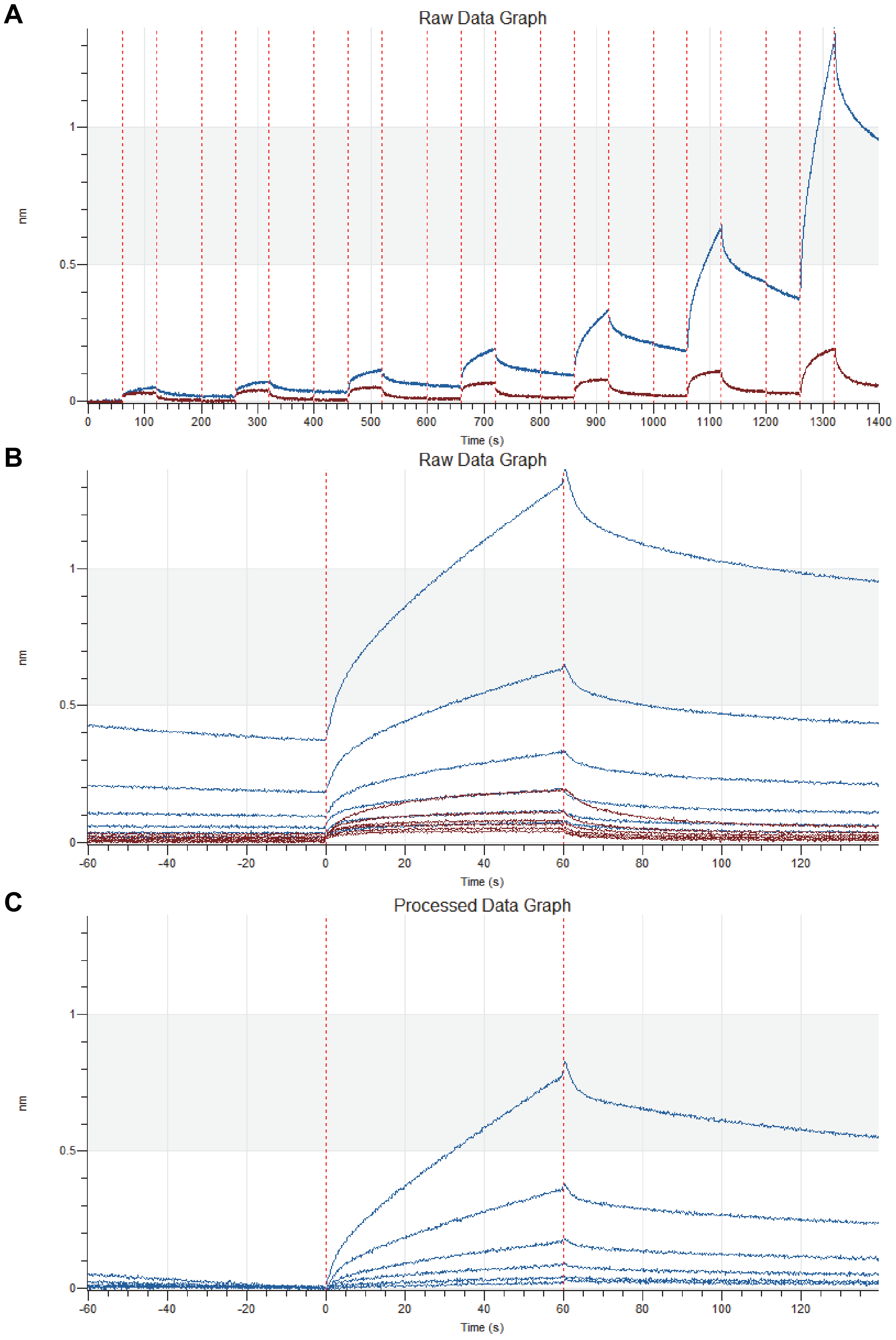

Supplementary Figure 10. BLI data processing for the interaction between free 601 DNA and PHF20L1 Tudor2.

(A) Raw sensorgrams recorded using free 601 DNA-immobilized biosensors (blue) and blank reference biosensors (red).

(B) Overlay of the raw sensorgrams at increasing concentrations of PHF20L1 Tudor2 prior to reference subtraction.

(C) Reference-subtracted binding curves generated by subtracting blank biosensor signals from free 601 DNA-immobilized biosensor signals. These processed curves were subsequently used for kinetic fitting and calculation of the binding parameters, including K_D, k_{on}, and k_{off}.

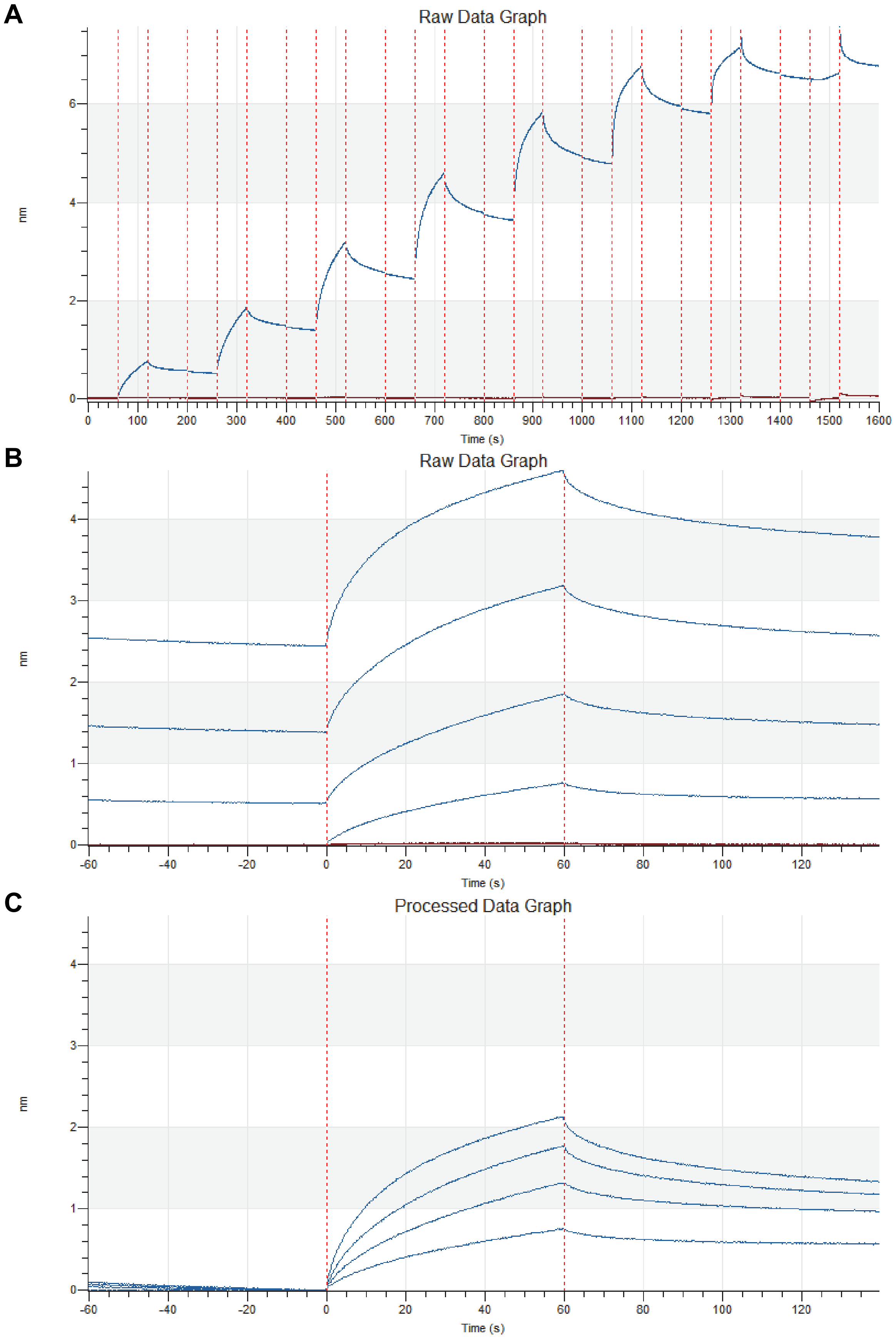

Supplementary Figure 11. BLI data processing for the interaction between H3K$_C$36me1-NCP and PHF20L1 Tudor1–2.

(A) Raw sensorgrams recorded using H3K$_C$36me1-NCP-immobilized biosensors (blue) and blank reference biosensors (red).

(B) Overlay of the raw sensorgrams at increasing concentrations of PHF20L1 Tudor1–2 prior to reference subtraction.

(C) Reference-subtracted binding curves generated by subtracting blank biosensor signals from H3K$_C$36me1-NCP-immobilized biosensor signals. These processed curves were subsequently used for kinetic fitting and calculation of the binding parameters, including K_D, k_{on}, and k_{off}.

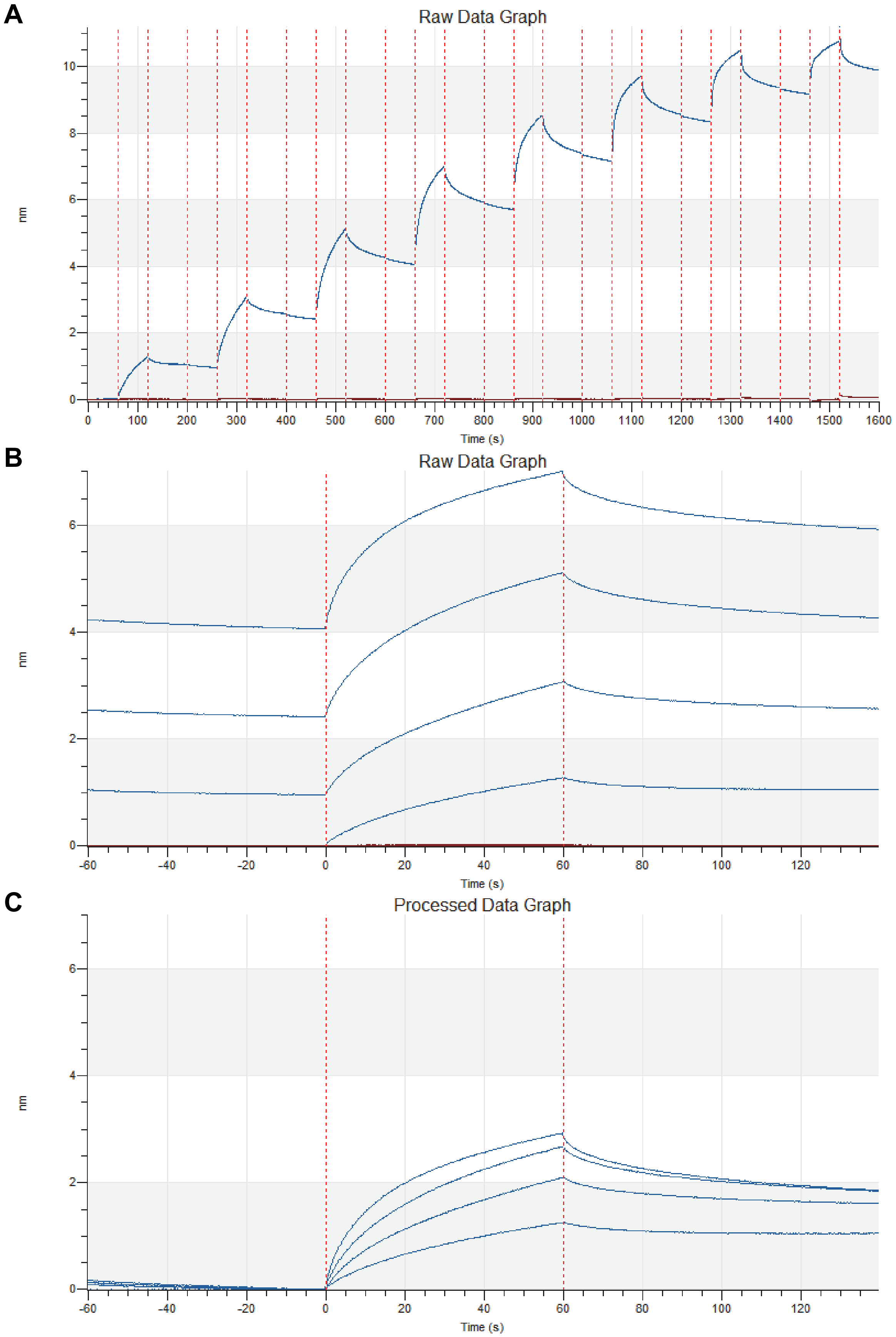

Supplementary Figure 12. BLI data processing for the interaction between $H4K_C20me2$-NCP and PHF20L1 Tudor1–2.

(A) Raw sensorgrams recorded using $H4K_C20me2$-NCP-immobilized biosensors (blue) and blank reference biosensors (red).

(B) Overlay of the raw sensorgrams at increasing concentrations of PHF20L1 Tudor1–2 prior to reference subtraction.

(C) Reference-subtracted binding curves generated by subtracting blank biosensor signals from $H4K_C20me2$-NCP-immobilized biosensor signals. These processed curves were subsequently used for kinetic fitting and calculation of the binding parameters, including K_D, k_{on}, and k_{off}.

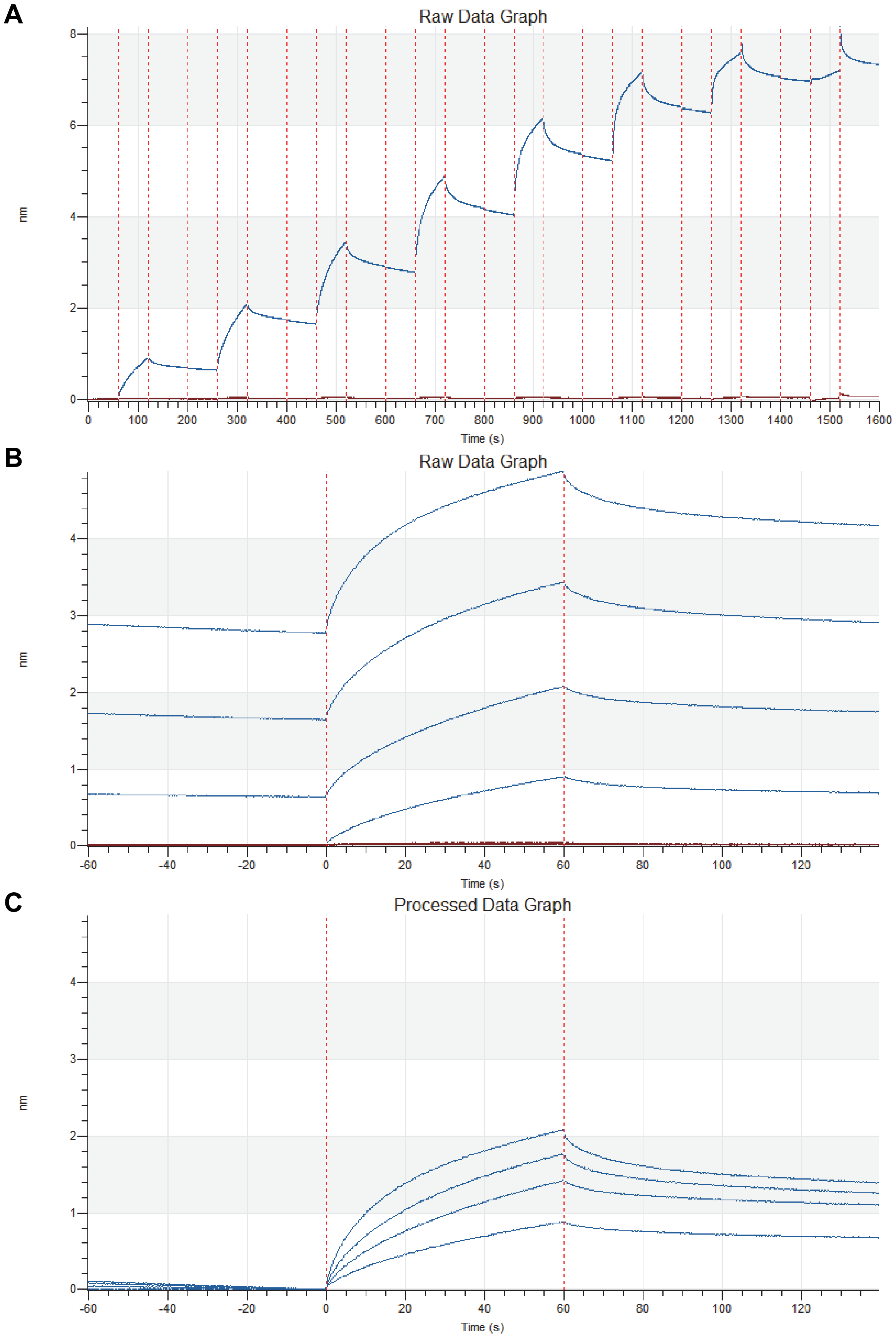

Supplementary Figure 13. BLI data processing for the interaction between H3K$_{C}$36me1/H4K$_{C}$20me2-NCP and PHF20L1 Tudor1–2.

(A) Raw sensorgrams recorded using H3K$_{C}$36me1/H4K$_{C}$20me2-NCP-immobilized biosensors (blue) and blank reference biosensors (red).

(B) Overlay of the raw sensorgrams at increasing concentrations of PHF20L1 Tudor1–2 prior to reference subtraction.

(C) Reference-subtracted binding curves generated by subtracting blank biosensor signals from H3K$_{C}$36me1/H4K$_{C}$20me2-NCP-immobilized biosensor signals. These processed curves were subsequently used for kinetic fitting and calculation of the binding parameters, including K_{D}, k_{on}, and k_{off}.

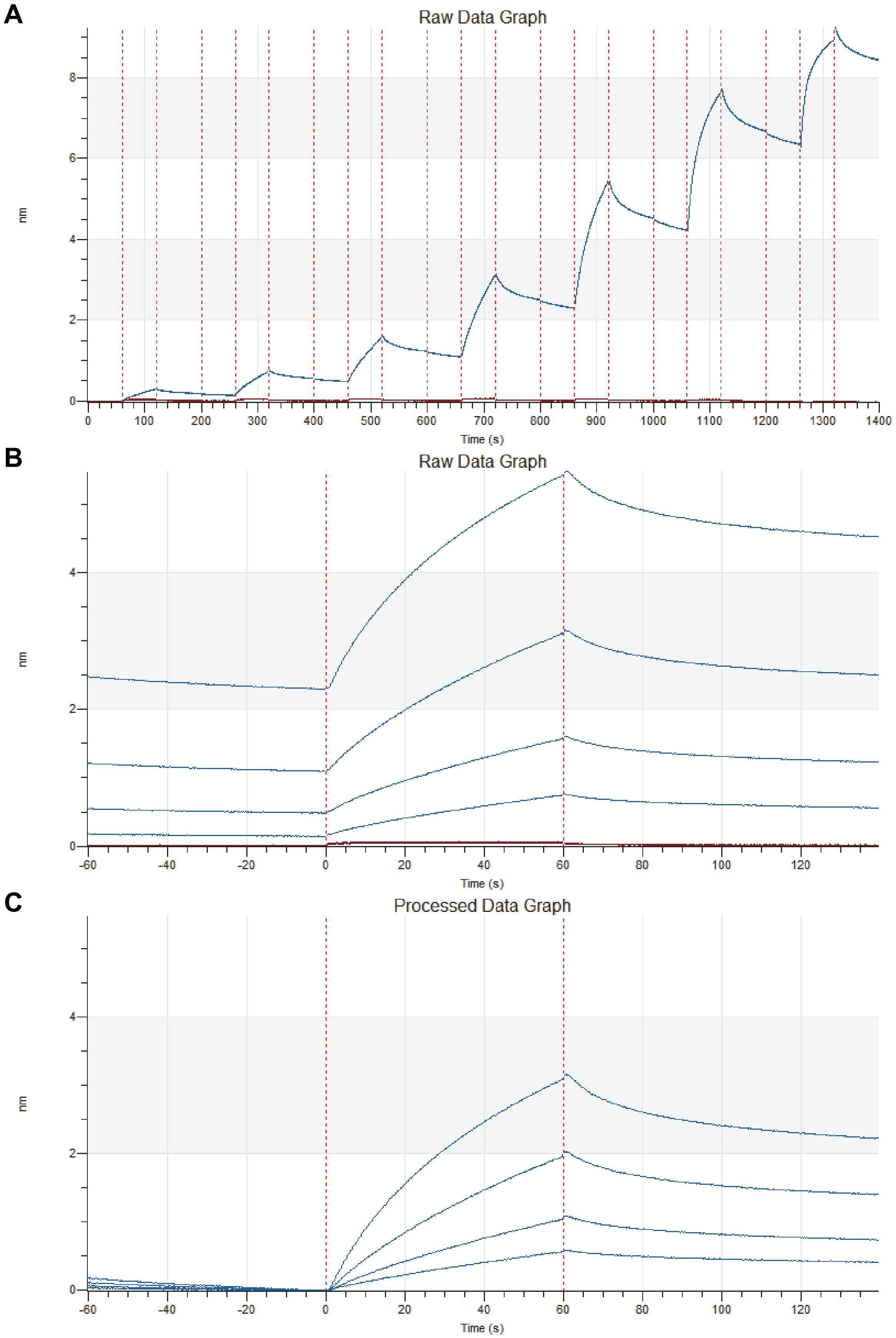

Supplementary Figure 14. BLI data processing for the interaction between free 601 DNA and PHF20L1 Tudor1–2.

(A) Raw sensorgrams recorded using free 601 DNA-immobilized biosensors (blue) and blank reference biosensors (red).

(B) Overlay of the raw sensorgrams at increasing concentrations of PHF20L1 Tudor1–2 prior to reference subtraction.

(C) Reference-subtracted binding curves generated by subtracting blank biosensor signals from free 601 DNA-immobilized biosensor signals. These processed curves were subsequently used for kinetic fitting and calculation of the binding parameters, including K_D, k_{on}, and k_{off}.
